# Genome-Wide Analysis of LRR-RLK Gene Family in Four *Gossypium* Species and Expression Analysis during Cotton Development and Stress Responses

**DOI:** 10.3390/genes9120592

**Published:** 2018-11-29

**Authors:** Ruibin Sun, Shaohui Wang, Dan Ma, Chuanliang Liu

**Affiliations:** 1 State Key Laboratory of Cotton Biology, Institute of Cotton Research, Chinese Academy of Agricultural Sciences, Anyang 455000, China; sunruibin@caas.cn (R.S.); wangshaohui@caas.cn (S.W.); madan@caas.cn (D.M.); 2 Zhengzhou Research Base, State Key Laboratory of Cotton Biology, Zhengzhou University, Zhengzhou 450066, China

**Keywords:** LRR-RLK family, *Gossypium*, expansion, phylogenetic analysis, gene expression profile, stress defense

## Abstract

Leucine-rich repeat receptor-like kinases (LRR-RLKs) have been reported to play important roles in plant growth, development, and stress responses. However, no comprehensive analysis of this family has been performed in cotton (*Gossypium* spp.), which is an important economic crop that suffers various stresses in growth and development. Here we conducted a comprehensive analysis of LRR-RLK family in four *Gossypium* species (*Gossypium arboreum*, *Gossypium barbadense, Gossypium hirsutum,* and *Gossypium raimondii*). A total of 1641 *LRR-RLK* genes were identified in the four *Gossypium* species involved in our study. The maximum-likelihood phylogenetic tree revealed that all the *LRR-RLK* genes were divided into 21 subgroups. Exon-intron organization structure of *LRR-RLK* genes kept relatively conserved within subfamilies and between *Arabidopsis* and *Gossypium* genomes. Notably, subfamilies XI and XII were found dramatically expanded in *Gossypium* species. Tandem duplication acted as an important mechanism in expansion of the *Gossypium* LRR-RLK gene family. Functional analysis suggested that *Gossypium* *LRR-RLK* genes were enriched for plant hormone signaling and plant-pathogen interaction pathways. Promoter analysis revealed that *Gossypium* *LRR-RLK* genes were extensively regulated by transcription factors (TFs), phytohormonal, and various environmental stimuli. Expression profiling showed that *Gossypium* *LRR-RLK* genes were widely involved in stress defense and diverse developmental processes including cotton fiber development and provides insight into potential functional divergence within and among subfamilies. Our study provided valuable information for further functional study of *Gossypium* *LRR-RLK* genes.

## 1. Introduction

Receptor-like protein kinases (RLKs) represent a large number of transmembrane kinases which perceive stimulus at the cellular surface and mediate the cellular signaling transduction via autophosphorylation and subsequent downstream phosphorylation for intercellular communication or response to the extracellular environment [1,2]. In land plants, RLKs form a large family and expand extensively [3,4,5]. Commonly, RLKs have an N-terminal extracellular domain (ECD) that varies in structure, a transmembrane domain (TM), and a relatively conserved cytoplasmic protein kinase catalytic domain (KD) [6]. The ECD region, which is thought to act as a ligand-binding site, has a variety of structural features, allowing it to interact with proteins, polysaccharides, lipids, and other ligands [4,7]. The leucine-rich repeat RLKs (LRR-RLKs) comprise the largest group of plant RLKs [7,8], which contain a varying number of leucine-rich repeat (LRR) kinases in the ECD region. The LRR is a 20–30 amino acid residue sequence motif, and appears to provide the structural framework for recognition of ligands [9]. The number and arrangement pattern of LRRs may vary among different LRR-RLKs, partly contributing to the diversity of LRR-RLKs. A comprehensive LRR-RLK analysis among a diversity of plants lineages classified plant LRR-RLKs into 19 subfamilies, and phylogenetic analysis demonstrated much of the diversity of plant LRR-RLKs was established in early land plants [10].

In plants, *LRR-RLK* genes play various important roles in plant development and response to biotic and abiotic stresses [10]. In terms of plant growth and development, the best characterized *LRR-RLK* gene member is *CLAVATA1* (*CLV1*) in *Arabidopsis*, which is involved in the development of shoot and flower apical meristem. By combining with receptor-like protein CLV2, the LRR-RLK CLV1 is dimerized and recognizes small secreted dodecapeptide CLV3 as the ligand to regulate expression of the downstream transcription factor *WUSCHEL* (*WUS*), which in return up-regulates the expression of *CLV3*, resulting in a feedback mechanism to adjust the meristem size [11,12,13]. The heterodimeric LRR-RLK complex BAK1/BRI1 initiates the brassinosteroid signaling cascade [14,15]. The one *LRR-RLK* gene *HAESA* (*HAE*) in *Arabidopsis* is involved in floral organ abscission [16], and *RPK1* and *TOAD2* encode LRR-RLKs required for proper embryo morphogenesis [17]. In addition, some LRR-RLKs are defense related. For instance, *Arabidopsis* FLS2 and EFR perceive bacterial antigens and mediate the defense against pathogens [18,19]. The rice *LRR-RLK* gene *Xa21* is an effective rice bacterial blight resistance gene [20]. As the metabolic pathway intertwines, some LRR-RLKs function on several aspects. For instance, somatic embryogenesis receptor-like kinases (SERKs) participate in the process of microsporogenesis and embryogenesis, and enhance acquisition of embryogenic competence in culture regeneration [21,22,23]. Meanwhile, recent research demonstrates that SERKs are indispensable in brassinosteroid signaling [24,25,26], including the rice *Os*SERK1 (encoded by the first identified *SERK* gene in rice) host defense response against fungal infection by mediating defense signaling transduction [23].

As LRR-RLKs have many functional roles, genome-wide identification and analysis of *LRR-RLK* genes has been carried out extensively. Based on KD sequence phylogeny and gene structure, at least 213 identified *LRR-RLK* genes in *Arabidopsis thaliana* were classified into 15 groups [3,8], 309 identified in *Oryza sativa* were classified into 5 groups [27], 379 identified in *Populus trichocarpa* were classified into 14 groups [28], 303 identified in *Brassica rapa* were classified into 15 groups [29], 467 identified in *Glycine max* were classified into 14 groups, and 234 identified in *Solanum lycopersicum* were classified into 10 groups [30]. To date, *LRR-RLK* genes have been identified and phylogenetically analyzed in more than 31 plant species [10,31]. However, no such analysis has been conducted on polyploid cotton (*Gossypium* spp.), except diploid species *Gossypium raimondii*, in which more than 300 *LRR-RLK* genes were identified [31,32].

Cotton, which comprises several *Gossypium* genus species, is an important economic crop, producing large amounts of natural fibers for the textile industry. Given the significant resource value of *Gossypium* genus, genome sequencing has been extensively promoted in *Gossypium*. To date, genomes of four *Gossypium* species including two widely cultivated allotetraploid *Gossypium* species (*Gossypium barbadense* and *Gossypium hirsutum*) and two diploid progenitor relatives species (*Gossypium arboreum* and *Gossypium raimondii*) have been sequenced and well-assembled [33,34,35,36,37,38], which laid a solid foundation for cotton research at the genomic level. For a long time, cotton suffered a variety of biotic and abiotic stresses during planting, and many efforts have been taken on the development of cotton fiber to improve the quality and yield. Considering the multifunction of *LRR-RLK* genes in plant defense response and development processes, in this study we conducted genome-wide identification and phylogenetic analysis of *LRR-RLK* genes on four genome-sequenced *Gossypium* genus species. In addition, the function and expression profiles of *Gossypium LRR-RLK* genes in several important developmental and stress response processes were analyzed. Our investigations provide insights into the evolution of *Gossypium LRR-RLK* genes and the roles of the LRR-RLK gene family in development and stress defense.

## 2. Materials and Methods

### 2.1. Identification of LRR-RLK Genes in Four Gossypium Genus Species

Up to now, four *Gossypium* species—*G. arboreum*, *G. barbadense*, *G. hirsutum*, and *G. raimondii*—have finished genome sequencing and assembly [34,35,36,37,38,39]. *G. hirsutum* and *G. barbadense* are the two most widely cultivated cotton species, both of which are allotetraploids and formed by inter-genomic hybridization of the A genome ancestral diploid and D genome ancestral diploid. *G. arboreum* (AA) and *G. raimondii* (DD) were recognized as species of progenitor relatives, whose progenitors were the putative A genome ancestor and D genome ancestor of *G. hirsutum* (AD1) and *G. barbadense* (AD2) [40,41]. Proteomes data of *G. arboreum*, *G. barbadense*, *G. hirsutum*, and *G. raimondii* were download from public databases (ftp://bioinfo.ayit.edu.cn/downloads; http://database.chgc.sh.cn/cotton/index.html; https://phytozome.jgi.doe.gov/ [42]), and the corresponding whole-genome gene annotations were downloaded as well. As LRR-RLKs are featured with KD domain, LRR domain, and TM domain, the corresponding Hidden Markov Model (HMM) models of KD including Pkinase (PF00069) and Pkinase_Tyr (PF07714), and HMM models of LRR including LRR_1 (PF00560), LRR_2 (PF07723), LRR_3 (PF07725), LRR_4 (PF12799), LRR_5 (PF13306), LRR_6 (PF13516), LRR_8 (PF13855), LRR_9 (PF14580)and LRV (PF01816), were downloaded from Pfam database (http://pfam.xfam.org/) [43] and provided as queries to conduct homologues search (*E*-value < 1 × 10^−10^) against the protein database of the four *Gossypium* species respectively by using HMMER 3.1b2 software [44]. The resulting hits that were obtained in both KD domain search results and LRR domain search results were collected for further filtering. To make sure that we get as close as possible to the whole LRR-RLKs, the amino acid sequences of *Arabidopsis* LRR-RLKs members reported by Shiu et al. [7] were retrieved from The Arabidopsis Information Resource (TAIR) database v10.0 (http://www.arabidopsis.org/) [45] and served as the query to perform a similarity search (*E*-value < 1 × 10^−5^, identity > 50%) against the protein database of the four *Gossypium* species using BLAST+ v.2.6.0 [46]. The sum total items of the HMMER search result and BLAST+ result was used for subsequent validation analysis. InterProScan v.5.24-63.0 [47] was used to confirm the presence of KD domain and LRR domain and other characteristic domains. TMHMM server v.2.0 (http://www.cbs.dtu.dk/services/TMHMM/) [48] and Phobius (http://phobius.binf.ku.dk/) [49] were used for TM domain prediction. When either TMHMM server or Phobius server indicated a TM domain, we decided it contained TM domain. Proteins that contained both KD domain, LRR domain, and TM domain were considered as LRR-RLKs.

### 2.2. Phylogenetic Analysis of Gossypium LRR-RLKs

*LRR-RLK* genes identified in four *Gossypium* species and previously reported in *Arabidopsis* [8] were involved in phylogenetic analysis. Complete amino acid sequences of identified LRR-RLKs were used to perform multiple sequence alignment by MUSCLE [50]. The maximum likelihood (ML) tree was constructed by FastTree 2 [51] with default arguments. Neighbor-joining (NJ) tree was constructed by MEGA 7 [52] with 1000 bootstrap.

### 2.3. Gene Structure Analysis

Exon-intron structure information of identified *LRR-RLK* genes was retrieved from the whole-genome gene annotations. LRR, TM, and KD domain coordinates were derived from InterProScan annotation results and TM domain prediction results. TBtools [53] was used to display the gene exon-intron structure and domain coding regions within the default parameters.

### 2.4. Genomic Distribution of LRR-RLKs and Tandem Duplication Identification

The genomic coordinates of genes were extracted from genome annotation release data, and then used to map *LRR-RLK* genes on chromosome by TBtools [53]. Tandem duplication of *LRR-RLK* genes were identified when genes belonged to the same LRR-RLK subfamily and separated by ten or less genes in a 200 kb distance.

### 2.5. Gene Ontology and Pathway Analysis of Gossypium LRR-RLKs

Gene Ontology (GO) and KEGG (Kyoto Encyclopedia of Genes and Genomes) pathway annotation information for *G. barbadense*, *G. hirsutum,* and *G. raimondii* were download from CottonFGD [54]. According to the gene functional annotation method used in CottonFGD, GO annotation of *G. arboreum* LRR-RLKs were conducted by InterProScan. KEGG Orthology was assigned to *G. arboreum* LRR-RLKs using KEGG Automatic Annotation Server (KAAS) [55] and then mapped to KEGG pathways. GO and KEGG pathway enrichment analysis was conducted by R package clusterProfiler v3.6.0 [56].

### 2.6. Promoter and Regulatory Analysis of Gossypium LRR-RLK Genes

The upstream 1.5 kb sequence of gene start codon was recognized and extracted as a promoter region. Promoter sequences of all *Gossypium LRR-RLK* genes were submitted to PlantCARE database [57] to predict potential cis-acting regulatory elements. Transcription factor (TF) binding sites were predicted by Binding site prediction tools on PlantTFDB 4.0 [58].

### 2.7. Gene Expression Profile Analysis of Gossypium LRR-RLK Genes

To investigate *Gossypium LRR-RLK* gene expression pattern during development and stress responses the following was conducted: RNA sequencing data of TM-1 ovule samples from −3, −1, 0, 1, 3, 5, 10, 20, and 25 days post anthesis (DPA), fiber samples from 5, 10, 20, and 25 DPA, and true leaves of the seedlings at different timepoints (1, 3, 6, and 12 h after treatment), after being treated with cold, heat, polyethylene glycol (PEG), and salt, were available at NCBI Sequence Read Archive (SRA) database (BioProject: PRJNA248163) [35]. Transcription level data were downloaded from CottonFGD [52] and analyzed. Transcriptomes of *G. barbadense* and *Gossypium hirsutum* in resistance response to *Verticillium dahlia* have been studied [59]. Two true leaf seedlings were inoculated with two different *V. dahlia* strains (highly aggressive strain V991 and intermediately aggressive strain D07038) by watering injured roots with *V. dahlia* spore suspension, while roots of control seedlings were watering with distilled water. For each treatment and control, plant samples from 24, 48, and 96 h after inoculation were mixed for sequencing. RNA sequencing data of response to infection were accessed from NCBI SRA (BioProject: PRJNA89721). The fragments per kilobase million (FPKM) value for each gene were computed to represent gene expression levels. An expression heatmap was drawn by R software package ComplexHeatmap (for *k*-means clustering) [60] and pheatmap (for hierarchy clustering) [61] based on log10-transformed FPKM values.

## 3. Results and Discussion

### 3.1. Identified LRR-RLK Genes of Four Gossypium Genus Species

A total of 298, 511, 515, and 317 *LRR-RLK* genes were identified in *G. arboreum*, *G. barbadense*, *G. hirsutum,* and *G. raimondii*, respectively. All of these identified *LRR-RLK* genes contained LRR domain, KD domain, and TM domain simultaneously, and the conserved protein domain arranged in the order of LRR-TM-KD from N-terminal to C-terminal (Figure S1). The number of *Gossypium LRR-RLK* genes accounted for 0.73%, 0.66%, 0.73%, and 0.85% of whole genome protein coding genes in each species, respectively. The proportions of *LRR-RLK* genes largely fit with the result of Liu et al. [10], which demonstrated 0.67–1.39% proportions in angiosperm species.

### 3.2. Phylogenetic Analysis and Gene Structure of Gossypium LRR-RLK Genes

**Figure 1:**
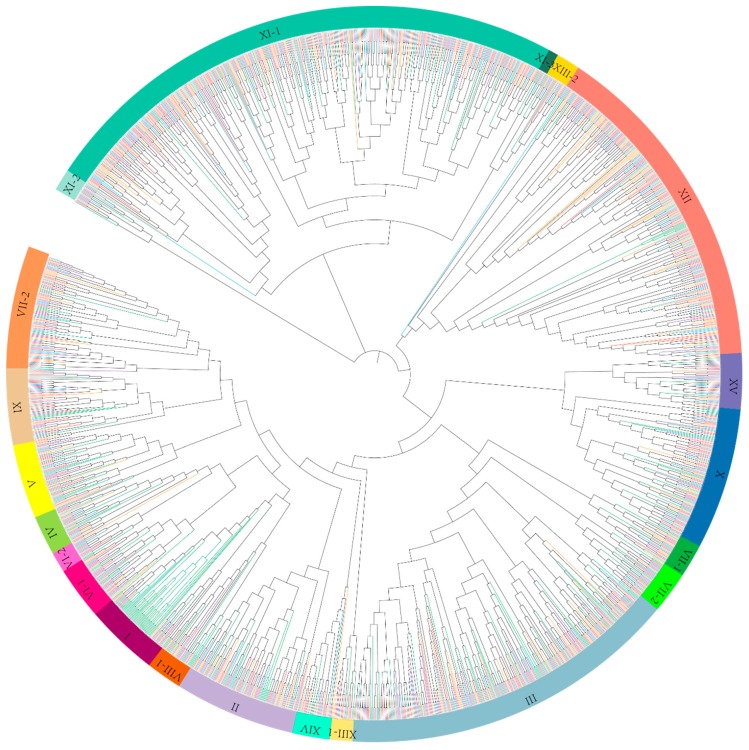
Phylogenetic tree of leucine-rich repeat receptor-like kinases (LRR-RLK) genes from four *Gossypium* species and *Arabidospsis thaliana*. The phylogenetic tree was constructed by maximum likelihood (ML) method based on kinase domain amino acid sequences of LRR-RLKs. All *LRR-RLK* genes were divided into 21 distinct clades, marked by bold curves with different colors. LRR-RLKs from *A. thaliana*, *Gossypium arboreum*, *Gossypium barbadense*, *Gossypium hirsutum,* and *Gossypium raimondii* were represented by branches colored within green, red, yellow, purple, and blue, respectively.

The amino acid sequences of 1641 *LRR-RLK* genes identified in this present study and previously reported 213 *A. thaliana LRR-RLK* genes were aligned (Supplementary data 1) for phylogenetic tree construction. Referring to the classification of *A. thaliana LRR-RLK* genes, the ML tree showed *LRR-RLK* genes from *Gossypium* were classified into 21 distinct clades (Figure 1, Supplementary data 2). To further validate the phylogenetic relationship of *LRR-RLK* genes in the ML tree, another tree based on the NJ method was constructed (Figure S2). Results showed that the topologies of both trees were somewhat different, while gene member assignment among different clades remained relatively stable. Therefore, the subfamilies classification in the ML tree was reliable and could be used for further analysis. Clades were named to correspond with subfamilies according to the nomenclature of *A. thaliana LRR-RLK* genes [8]. Most of LRR-RLK subfamilies of *Gossypium* were consistent with *A. thaliana*, while subfamilies VI, VII, VIII, and XI were further divided into VI-1 and VI-2, VII-1 and VII-2, VIII-1 and VIII-2, XI-1, XI-2, and XI-3, respectively. The detailed classification of *Gossypium* and *A. thaliana LRR-RLK* genes was described in Table S1. Overall, subfamilies III, XI, and XII showed the highest number of LRR-RLKs. Meanwhile, in terms of *A. thaliana*, the majority of LRR-RLKs were distributed in subfamilies I, III, XI and (Table 1, Figure 1).

**Figure 2:**
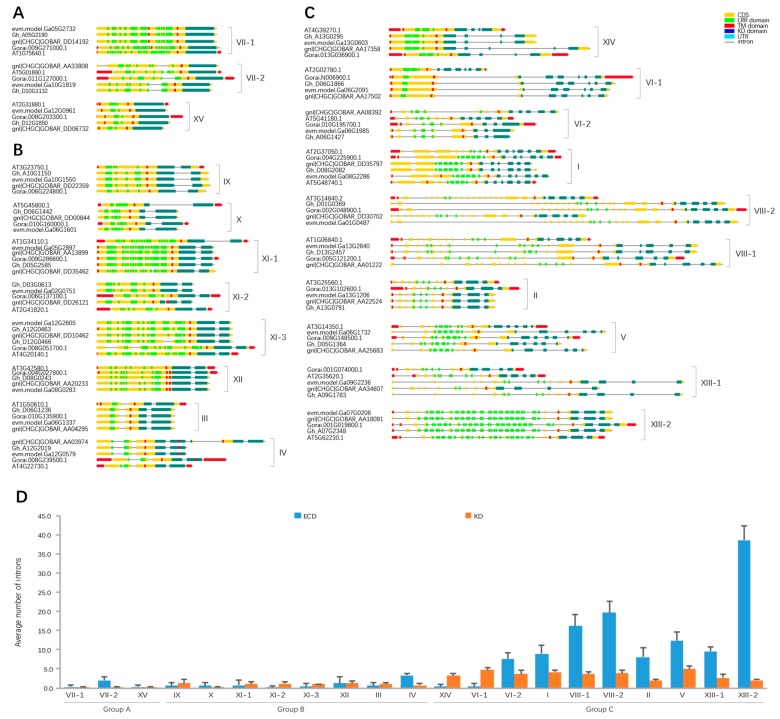
Exon-intron structures of representative *LRR-RLK* genes of each subfamily from four *Gossypium* species and *A. thaliana*. LRR (leucine-rich repeat), KD (kinase catalytic domain), and TM (transmembrane) domain coding regions were marked on exons by different colored rectangles. Based on the exon-intron structures of KD domain, *Gossypium LRR-RLK* genes were classified into Group A (**A**), Group B (**B**), and Group C (**C**). The number of introns in ECD (extracellular domain) and KD domain were counted and then average values were computed for each subfamily (**D**). The error bar on the column represented standard deviation of the subfamily.

Gene exon-intron structures and characteristic domain organizations of *Gossypium LRR-RLK* genes were investigated (Figure S1). For each subfamily, gene structures of representative genes from each species were displayed and compared. The number of introns in the ECD region varied both within and between subfamilies, while the number of introns in the KD domain were relatively constant (Figure 2D). Variable ECD regions might help LRR-RLKs to perceive diverse environmental stimuli. As the KD domain is more conserved than the LRR domain in LRR-RLK family genes [10,31], we classified the *Gossypium LRR-RLK* genes into three groups based on exon-intron organization of KD domain (Figure 2A–C). *LRR-RLK* genes in Group A, which contained subfamilies VII-1, VII-2, and XV, had KD domains located on an integral exon (Figure 2A), while KD domains of *LRR-RLK* genes in Group B, containing subfamilies III, IV, IX, X, XI-1, XI-2, XI-3, and XII, were separated by one intron (Figure 2B). In Group C, containing subfamilies I, II, V, VI-1, VI-2, VIII-1, VIII-2, XIII-1, XIII-2, and XIV, KD domains of *LRR-RLK* gene members were separated into 3–6 exons by introns. Meanwhile, the ECD regions of Group C genes (except for subfamilies XIV and VI-1 members) showed highly discrete distributed exons (Figure 2C). As a result, the LRR domains of Group C gene members were distributed in many different exons. Conversely, the majority of genes’ LRR domains in Group A and B were located in an integral exon. Referring to subfamily division based on the ML tree, exon-intron structures of *LRR-RLK* genes were variable between different subfamilies, while *LRR-RLK* genes in the same subfamily showed comparable gene structures. This indicated that gene structures were relatively conserved within each subfamily, implying that evolutionary relationships of these genes coincided with the ML tree. For each subfamily, gene structures of *LRR-RLK* genes from *A. thaliana* and four *Gossypium* species were almost identical, suggesting that exon-intron structures of *LRR-RLK* genes were relatively conserved between *A. thaliana* and *Gossypium*. However, there were some exceptions. In subfamily IX, there was a subclade containing five gene members (*GOBAR_DD21842*, *evm.model.Ga14G2553*, *Gorai.010G147700*, *GOBAR_AA01355*, and *evm.model.Ga06G1442*) which showed different exon-intron structures, in which the KD coding regions were divided into five exons (except for *GOBAR_AA01355*, which has a truncated KD domain), which differed from the representative two-exon KD (kinase catalytic domain) domain of subfamily IX (Figure S1). The phylogenic tree showed that this subclade was distinctly separated from other subfamily IX members with strong bootstrap support (0.98) (Supplementary data 2), further implying the consistency between the phylogenic tree and gene structure. Some *LRR-RLK* genes lost all introns compared to their close homologues, including 8 subfamily XI-1 gene members (*Gh_D11G2499*, *Gh_A07G0162*, *Gh_D09G1175*, *GOBAR_AA08625*, *GOBAR_AA07945*, *Gh_A06G0294*, *Gh_D09G0173*, and *Gh_D09G0176*), 6 subfamily III gene members (*GOBAR_DD29294*, *GOBAR_AA04771*, *Gh_D05G0350*, *GOBAR_AA35240*, *GOBAR_DD18442*, and *Gh_A05G0258*) and 3 subfamily XII gene members (*GOBAR_DD24038*, *Gh_D05G3552*, *Gh_D10G2231*). These genes could be the retrotransposed genes. Retrotransposition is an important mechanism for genome expansion and is ubiquitous in plants [62]. For the LRR-RLK family in *Gossypium*, all these retrotransposed genes were found in the three largest subfamilies, suggesting that retrotransposition may contribute to the high gene numbers of these subfamilies to some degree.

### 3.3. Dramatic Expansion of LRR-RLK Subfamilies XI and XII in Gossypium Species

Statistics of *LRR-RLK* gene distribution among different subfamilies was conducted in both *Gossypium* species and *A. thaliana*. As shown in the phylogenetic tree (Figure 1), some subfamilies exhibit linage specific expansion. For example, in subfamily XII clade, the overwhelming majority of members belong to *Gossypium*, with only 7 of the total 282 members belonging to *A. thaliana*. Another example is the subfamily I clade, which is mainly (41 out of 61) composed of *A. thaliana* LRR-RLKs (Table 1, Figure 1). An uncoordinated proportion of *LRR-RLK* members between *Gossypium* and *A. thaliana* in these subfamilies suggested different expansion patterns of *LRR-RLK* genes between them.

**Figure 3:**
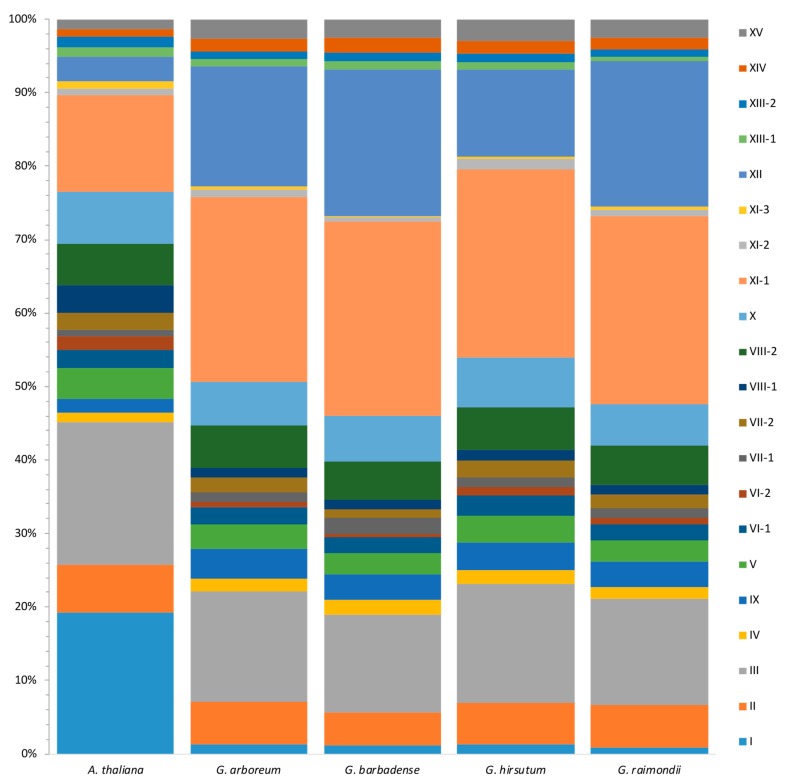
Comparison of *LRR-RLK* gene distribution among different subfamilies between *A. thaliana* and *Gossypium*. For each species, all the LRR-RLKs were divided into 21 subfamilies, represented by rectangles within different colors. The area of each rectangle within a specific color represented the proportion of the corresponding subfamily.

To further investigate the expansion pattern of different linages, we compared the distribution of *LRR-RLK* genes among different subfamilies between *A. thaliana* and *Gossypium*. In consideration of the genome size difference among different species, the proportion of each subfamily was compared instead of member number of each subfamily. In general, *LRR-RLK* genes showed similar proportion in most subfamilies between *Gossypium* and *A. thaliana*, while *Gossypium LRR-RLK* genes had a significantly smaller proportion of subfamily I distribution, and a larger proportion of subfamily XI and XII than *A. thaliana* (Figure 3). There were 41 LRR-RLK I members in *A. thaliana*, accounting for 19.2% of all *A. thaliana LRR-RLK* genes, while the counterpart percentage was 0.9–1.4% in *Gossypium* species. *A. thaliana* had about 13 times more LRR-RLK I members compared to *Gossypium* species. On the contrary, *Gossypium* species showed 26.5–27.4% and 11.8–20.0% of LRR-RLKs distributed in subfamily XI and XII, respectively. The proportions were about two to four times more than in *A. thaliana*, in which the corresponding percentage were only 15.0% and 3.3%.

As the last common ancestor of angiosperms (LCAA) was estimated to contain about seven LRR-RLK subfamily I members, *A. thaliana* LRR-RLK I subfamily shows dramatic expansion due to whole-genome duplication (WGD) of *Brassicaceae* [31]. The significant expansion of subfamily I in *A. thaliana* made it to be the largest subfamily, while a slight reduction of this subfamily was found in diploid *Gossypium*, which perhaps suggested gene loss during evolution. With respect to LRR-RLK subfamily XI and XII, compared with LCAA, all four *Gossypium* species exhibited significant expansion in these two subfamilies. As described in a previous study [31], the LRR-RLK XI subfamily almost kept stable member numbers in most angiosperms, as verified by *A. thaliana* in this present study, while in *Gossypium*, subfamily XI expands dramatically (predominantly in XI-1), making it to be the largest LRR-RLK subfamily (accounting for about 25% of the total *LRR-RLK* genes). Current knowledge suggests that many of the *A. thaliana LRR-RLK* genes that fall into XI were involved in plant organ and tissue development. For example, *RGFR* genes are involved in root growth and development. *CLV1* and *BAM* are involved in shoot and floral meristem development and function. *PXY* is involved in vascular-tissue development. *HAE* and *HSL* control floral organ abscission. *IKU* and *GSO* are involved in embryo development. Besides some other *A. thaliana* XI gene members, such as *PEPR1* and *PEPR2* which are defense and stress response-related, expanded subfamily XI might help *Gossypium* to defend against stresses. Interestingly, according to the phylogenic tree, the one large clade of subfamily XI-1 containing *AT4G08850* (*MIK2*) and *AT1G35710* was almost entirely comprised of *Gossypium* LRR-RLK members (Figure 1), suggesting that the orthologs of these two *A. thaliana* XI genes were largely expanded in *Gossypium*, which largely accounted for the expansion of subfamily XI-1 in *Gossypium*. According to previous studies [28,31,63], the dramatic expansion of homologues of *AT4G08850* (*MIK2*) and *AT1G35710* was also detected in many other plant species, such as *P. trichocarpa*, *G. max*, *Malus × domestica*, and *Prunus persica*, though the corresponding clade was sometimes assigned a different name. It has been reported that *MIK2* acts as a key component of the male receptor heteromer in the pollen tube cell perceiving female attractants in plants [64]. Other studies showed that *MIK2* is stress defense related. *MIK2* up-regulates expression in the root under salt exposure, and enhances rosette growth maintenance under salt stress conditions [65,66]. In cell wall damage response-processes, MIK2 acts as an important sensor and regulator, involved in response to abiotic and biotic stresses [67]. Little information about the function of *AT1G35710* gene was known, however, a transcriptome study found that the *AT1G35710* gene was repressed by linolenic acid, which is a precursor of the phytohormone jasmonic acid (JA) and launched a set of defense responses to pathogen attacks [68], suggesting that *AT1G35710* may be involved in defense response. As homologues within the same cluster may have similar functions, we supposed that the expansion of subfamily XI-1 in *Gossypium* may enhance defense of cotton against diverse environmental stimuli and stresses. Likewise, subfamily XII expands greatly in *Gossypium*. It is already known that the expansion of LRR-RLK XII is extensive in many different species [31]. Most genes in subfamily XII are involved in biotic and abiotic stress response, so we supposed that the expansion of LRR-RLK XII helps in perceiving and adapting to diverse environments. All of these results were consistent with previous findings that lineage-specific expanded *LRR-RLK* genes predominantly belong to subgroups involved in environmental interactions [31].

As allotetraploid species *G. hirsutum* and *G. barbadense* are known to have derived from hybridization of diploid ancestors of *G. arboreum* and *G. raimondii* [33,34,41], the number of LRR-RLK members in each subfamily between the two allotetraploid species were compared. In general, *G. hirsutum* and *G. barbadense* have similar proportions in terms of most of the subfamilies. However, when regarding subfamily XII, *G. barbadense* showed a significantly higher proportion than *G. hirsutum* (Figure 3). In further detail, *G. barbadense* had 102 *LRR-RLK* genes assigned into subfamily XII, as there were only 61 LRR-RLK subfamily XII members in *G. hirsutum*. As most subfamily XII members are defense-related, we supposed that more subfamily XII LRR-RLK members may confer better resistance in *G. barbadense* than *G. hirsutum* to some extent. We supposed that *G. barbadense* is more likely to retain copies of LRR-RLK subfamily XII gene members from diploid ancestors, while *G. hirsutum* tends to lose some copies. Different retain/loss models of duplicates after polyploidization between *G. hirsutum* and *G. barbadense* may be due to adaptation under different environments and different selection pressures.

### 3.4. Genomic Distribution and Gene Duplication of LRR-RLK Genes in Gossypium

The genomic distribution of identified *LRR-RLK* genes form four *Gossypium* species was displayed respectively (Figure S3). Most of the *LRR-RLK* genes were mapped on chromosomes, with only 11(3.7%), 19(3.7%), 40(7.8%), and 4(1.3%) *LRR-RLK* genes from *G. arboreum*, *G. barbadense*, *G. hirsutum*, *G. raimondii* being located on scaffolds, respectively. The *LRR-RLK* genes were distributed in all chromosomes but unevenly across different chromosomes (Table S2). The distribution across different chromosomes was comparative in two polyploid species, chromosome A5, A10, D5, and D10 containing the highest proportion of *LRR-RLK* genes in both *G. barbadense* and *G. hirsutum*. The comparable genomic distribution pattern of this large gene family between *G. barbadense* and *G. hirsutum* suggested the close phylogenic relationship of these two allotetraploid species. However, the distribution across different chromosomes was quite different between two diploid species *G. arboreum* and *G. raimondii* (Figure S3).

Mapping *LRR-RLK* genes on chromosomes allows us to detect gene duplication. In our analysis, *LRR-RLK* genes which fell into the same subfamily and were separated by 10 or less genes in a 200 kb chromosomal distance were recognized as a tandem duplication set [5,28]. There were 26, 47, 33, and 25 tandem duplication sets involving 79, 146, 92, and 96 tandem duplicates found in *G. arboreum*, *G. barbadense*, *G. hirsutum,* and *G. raimondii,* respectively. Gene numbers contained in each tandem duplication set ranged from 2 to 11. Furthermore, tandem duplication occurred unevenly among different subfamilies (Table 2). The dramatically expanded subfamilies XI and XII contained the majority of all tandem duplication sets (73.1%, 78.7%, 69.7%, and 72.0% for *G. arboreum*, *G. barbadense*, *G. hirsutum,* and *G. raimondii,* respectively) and tandem duplicated genes (78.5%, 84.2%, 75.0%, and 81.3% for *G. arboreum*, *G. barbadense*, *G. hirsutum*, and *G. raimondii,* respectively). Subfamily VIII-2 contained about 14.5% of tandem duplication sets and 11.7% of tandem duplicated genes, corresponding to somewhat of an expansion of subfamily VIII-2. Other subfamilies (II, III, VII-1, and IX) contained a few sets of tandem duplication. No tandem duplication was found in subfamilies I, IV, V, VI-1, VI-2, VII-2, VIII-1, X, XI-1, XI-3, XIII, XIV, and XV) (Table 2 and Table S3). When concerning the two dramatically expanded subfamilies XI and XII, we found that about 40% of LRR-RLK subfamilies XI members and about 60% of LRR-RLK XII members were involved in tandem duplication (Table S3), implying that tandem duplication played an important role in vast expansion of these subfamilies. Furthermore, just as in subfamilies XI and XII, about half of the LRR-RLK subfamily VIII-2 members were derived from tandem duplication (Table S3), suggesting the same important role of tandem duplication in expansion of subfamily VIII-2. We deduced that tandem duplication existed extensively and acted as an important expansion mechanism in expanded LRR-RLK subfamilies.

### 3.5. Functional and Pathway Annotation Analysis of Gossypium LRR-RLK Genes

**Figure 4:**
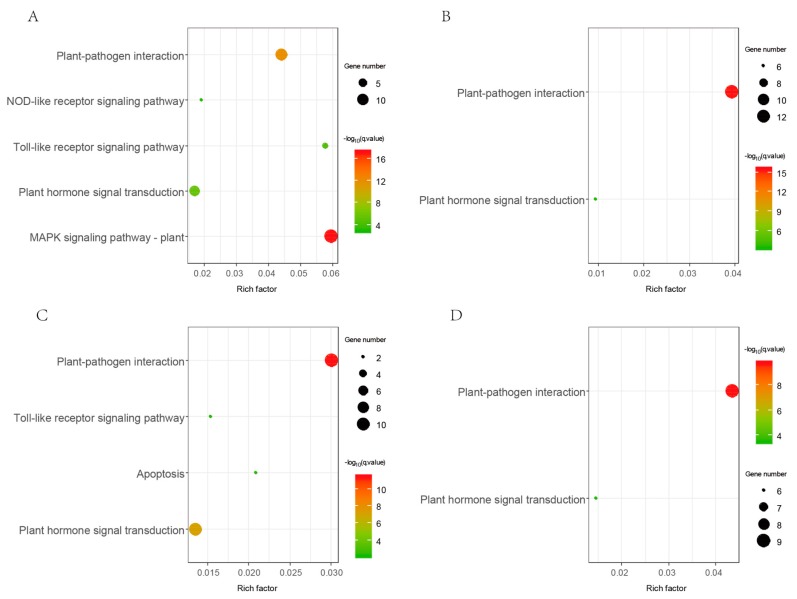
KEGG (Kyoto Encyclopedia of Genes and Genomes) pathway enrichment result of *Gossypium LRR-RLK* genes. Results of *G. arboreum*, *G. barbadense*, *G. hirsutum,* and *G. raimondii* were shown by (**A**–**D**) respectively.

Gene ontology annotation information was available from public *Gossypium* databases. We conducted GO enrichment on all *LRR-RLK* genes from *G. arboreum*, *G. barbadense*, *G. hirsutum,* and *G. raimondii*. Results showed that the molecular function of “protein kinase activity”, “protein binding”, “ATP binding”, and biological processes of “protein phosphorylation” were significantly enriched in *Gossypium LRR-RLK* genes (Figure S4). The GO enrichment results were confirmed with the basic kinase attributes of LRR-RLKs. KEGG pathway enrichment analysis showed that “plant pathogen interaction” and “plant hormone signal transduction” were significantly enriched in all four *Gossypium* species (Figure 4). Furthermore, “toll-like receptor signaling” and “NOD-like receptor signaling pathway” were found to be enriched in *G. arboreum* and *G. hirsutum*. These results suggested that the functional roles of *LRR-RLK* genes in plant development and defense may be largely mediated by related signaling.

### 3.6. A cis-Acting Regulatory Analysis of Gossypium LRR-RLK Genes’ Promoters

We found that cis-acting regulatory elements in the promoter regions (1.5 kb sequence upstream of start codon) of *Gossypium LRR-RLK* genes were detected by searching PlantCARE database. Phytohormone, stresses defense, and cell cycle related cis-acting regulatory elements were widespread in the promoter regions of *Gossypium LRR-RLK* genes (Table 3 and Table S4). More than 90% of *Gossypium LRR-RLK* genes had water stress, drought stress, and light response cis-acting regulatory elements in their promoters. Anoxic stresses response, wounding, and pathogen response cis-acting regulatory elements were found in promoter regions of about 80% of *Gossypium LRR-RLK* genes. Additionally, more than half of *Gossypium LRR-RLK* genes had heat, osmotic stress, low pH, nutrient starvation, ethylene (ETH), and abscisic acid (ABA) response cis-acting regulatory elements in their promoter regions. Jasmonate response elements existed in many *Gossypium LRR-RLK* genes’ promoters (50.7%, 42.5%, 44.7%, and 48.3% of *G. arboreum*, *G. barbadense*, *G. hirsutum,* and *G. raimondii LRR-RLK* genes, respectively). Salicylic acid (SA) and gibberellin response elements were also found in about 40% of *Gossypium LRR-RLK* genes’ promoters. Auxin response elements were found in about 30% of *Gossypium LRR-RLK* genes’ promoters. TC-rich repeats (defense and stress response) and LTR (low temperature response) elements were found in promoters of about 30% of *Gossypium LRR-RLK* genes. More than 45% of *Gossypium LRR-RLK* genes had cell cycle and cell proliferation related elements in promoter regions. Some (about 5%) *Gossypium LRR-RLK* genes had heavy metal ions response-related cis-acting regulatory elements. The cis-acting regulatory elements analysis revealed that the expression of *Gossypium LRR-RLK* genes was extensively regulated by phytohormone and other diverse abiotic and biotic environmental signals, implying the important roles of *Gossypium LRR-RLK* genes in stresses defense and development. Compared with *A. thaliana*, there were more heat, osmotic stress, low pH, nutrient starvation, and ETH response cis-acting elements but less ABA, JA, and auxin response cis-acting elements in promoter regions of *Gossypium LRR-RLK* genes. We further conducted enrichment analysis on each subfamily to investigate the probable over-represented cis-acting regulatory elements. As a result, circadian response element and Cd (cadmium) response element were overrepresented in subfamily XV and subfamily VIII-2 of *G. barbadense*, respectively. Cold response element was overrepresented in subfamily IX of *G. hirsutum*.

**Figure 5:**
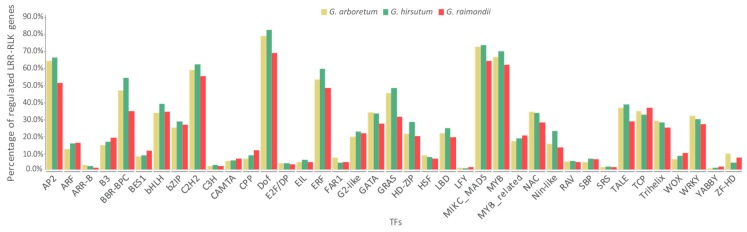
Statistics of *Gossypium LRR-RLK* genes regulated by different families of transcription factors (TFs) (genes with TF binding sites were considered to be regulated by TFs).

Transcription factors (TFs) play key roles in many cellular and biological processes by regulating expression of corresponding target genes. To investigate the possible regulation relationship between TFs and *Gossypium LRR-RLK* genes, TF binding sites were predicted by an online tool—binding site prediction on PlantTFDB [58]. Results showed that *Gossypium LRR-RLK* genes could be regulated by 39 TF families (Figure 5, Table S5). Dof, MIKC_MADS, MYB, AP2, C2H2, and ERF were the most widely functioning TF families and could regulate the majority of *Gossypium LRR-RLK* genes. Most of the top TF families were implicated in various aspects of plant development, hormonal signal transduction, plant defense, and stresses response, suggesting that *Gossypium* LRR-RLKs might participate in diverse plant development and stress defense processes by TF mediated regulation.

### 3.7. Gene Expression of Gossypium LRR-RLKs during Developmental and Stress Defense Processes

**Figure 6:**
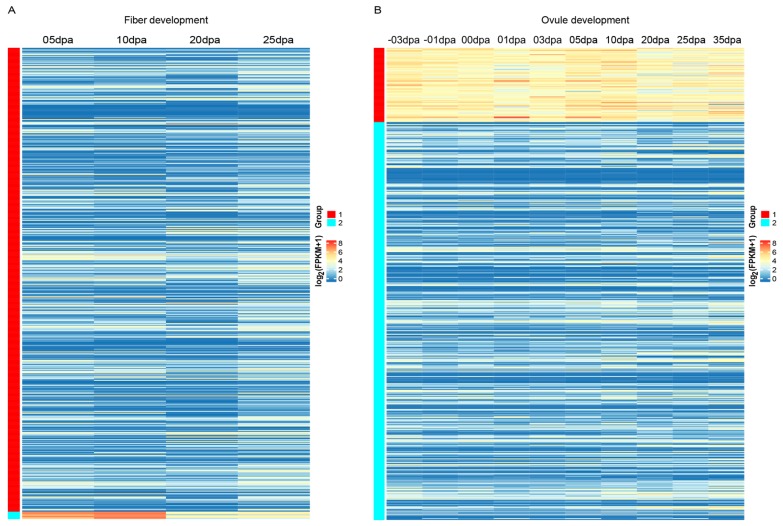
Expression patterns of *G. hirsutum LRR-RLK* genes in fiber and ovule development. Genes were clustered by the *k*-means method.

As the functional analysis showed the important roles of *Gossypium* LRR-RLKs in diverse developmental and defense processes, the expression profilers of *Gossypium* LRR-RLKs in several important developmental (fiber development, ovule development) and biotic (Verticillium wilt) stress defense and abiotic (cold, hot, drought, and salt) stress defense processes were investigated. Fiber development is an important process in cotton biology, based on the transcription dynamics of *LRR-RLK* genes during fiber development. The *k*-means clustering result showed that *G. hirsutum LRR-RLK* genes were clustered into two groups. The majority of Group 1 gene members showed relatively lower or moderate expression throughout the fiber development, while gene members in Group 2 were obviously actively expressed. Genes in Group 2 were highly expressed at 5 and 10 dpa (day post anthesis) stages, followed by down-regulation during 20 and 25 dpa (Figure 6A). In cotton fiber development, fiber cell initials start at 0 dpa and elongate subsequently from 0–18 dpa. Additionally, 20 dpa is commonly considered as the key stage of transition to secondary cell wall growth, followed by dehydration and maturation after 30 dpa [69,70,71]. Expression patterns of Group 2 genes showed high correlation with the elongation phase of fiber development, suggesting that these genes were extensively involved in the rapid elongation of fiber. There were eight *LRR-RLK* genes assigned into Group 2 (three subfamily IX members: *Gh_D09G1268*, *Gh_A13G0257*, and *Gh_D13G0274*; two subfamily V members: *Gh_A10G0460* and *Gh_D10G0477*; two subfamily III members: *Gh_A11G1546* and *Gh_D11G3486*; and another subfamily I member gene *Gh_A07G1471*). Except for *Gh_A07G1471*, the other Group 2 members belong to 3 homologous pairs. All three subfamily IX members in Group 2 have orthologous gene in *A. thaliana* known as *TMK3*, which is reported to play an essential role in plant growth mediated by regulation of cell expansion and auxin signaling [72]. Therefore, we suggested that these three *LRR-RLK* genes would likely have contributed to the elongation of cotton fiber, given that the elongation of fiber is almost a longitudinal expansion of singular fiber cells. The two homologous subfamily III members in Group 2 have the orthologs of *RLK1* in *A. thaliana*, as two homologous subfamily V members in Group 2 are orthologs of *A. thaliana SRF6*. Further investigation of expression profiles in different tissues showed that all eight Group 2 genes were predominantly expressed in early-period fiber, especially the developmental stage of 5–10 dpa [73], further implying the role of these genes in fiber elongation. Cloning and functional identification of these genes in fiber development would prove a worthy finding.

In the process of ovule development, all *G. hirsutum LRR-RLK* genes were divided into two groups according to *k*-means clustering. Group 1 contained 81 *LRR-RLK* genes that showed high expression at almost all stages of ovule development (Figure 6B), implying their important role in ovule development. Most of the Group 1 genes belonged to subfamily III, II, and XI-1. Protein orthologs of Group 1 genes contained several members involved in embryo and gamete development (such as BAM, BAK1, EMS1, RPK2, TOAD2, and ERECTA) and members involved in hormone signaling (such as RGI3 and BRI1). Moreover, the 6 *LRR-RLK* genes (*Gh_A10G0460*, *Gh_D10G0477*, *Gh_A11G1546*, *Gh_D11G3486*, *Gh_A13G0257* and *Gh_D13G0274*) that participated in fiber development as described above were also assigned to Group 1 in the ovule development *k*-means clustering. This implied the versatility of single *G. hirsutum LRR-RLK* genes, which might participate in multiple developmental processes.

**Figure 7:**
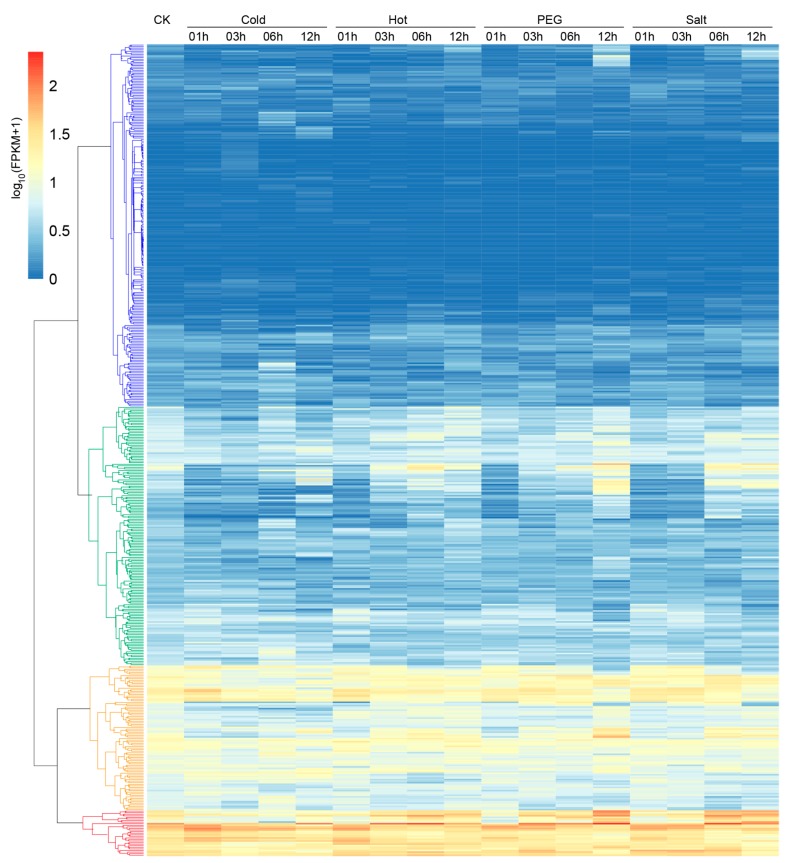
Expression patterns of *G. hirsutum LRR-RLK* genes in response to diverse abiotic stresses (cold, hot, drought simulated by polyethylene glycol (PEG), and salt). Hierarchical clustering analysis classified genes into four distinct groups (colored by blue, green, orange, and red, respectively).

Cotton is inevitably threatened by diverse abiotic stresses during its growth and development. Therefore, expression profiles of *G. hirsutum LRR-RLK* gene responses to cold, hot, drought, and salt stresses were analyzed. The *G. hirsutum LRR-RLK* genes were clustered in four groups based on their expression profile responses to different abiotic stresses (Figure 7). Different gene sets responded to different abiotic stimulates by changing expressions in a temporal manner. About half of *G. hirsutum LRR-RLK* genes depicted low expression during the stress treatments, as shown in Group blue which contains *LRR-RLK* genes belonging to almost all subfamilies except for subfamily VIII-1. The majority of *LRR-RLK* genes in Group green, which consists of *LRR-RLK* genes from almost all subfamilies except for subfamily XI-3, showed relatively lower expression levels in control. Most were down-regulated in response to the four abiotic stresses compared to control, while some members subsequently up-regulated at later response stages. Genes in Group orange and Group red showed relatively higher expression level in control. Most of Group orange’s genes down-regulated under all stresses, with different genes down-regulating at different stages of stress responses, suggesting diverse mechanisms of LRR-RLK gene response to abiotic stresses. Group orange consisted of *LRR-RLK* genes from almost all subfamilies except for subfamilies I, XI-3, and XIV. Genes in Group red stayed highly expressed during all stress response processes. Most of them up-regulated at early stages of cold and hot exposure but at later stages of PEG and salt stress treatments, implying that genes in Group red might act as positive regulators in abiotic stress responses. There were 29 *LRR-RLK* genes belonging to 9 different subfamilies (IX, V, VII-2, VIII-2, X, XI-1, XIII-1, XIV, and XV) in Group red, with subfamily XI-1 accounting for the largest proportion (12 genes). These results implied that *Gossypium LRR-RLK* genes were multi-functional, play important roles in multiple abiotic stress responses, and might help cotton to adapt to diverse abiotic environments.

**Figure 8:**
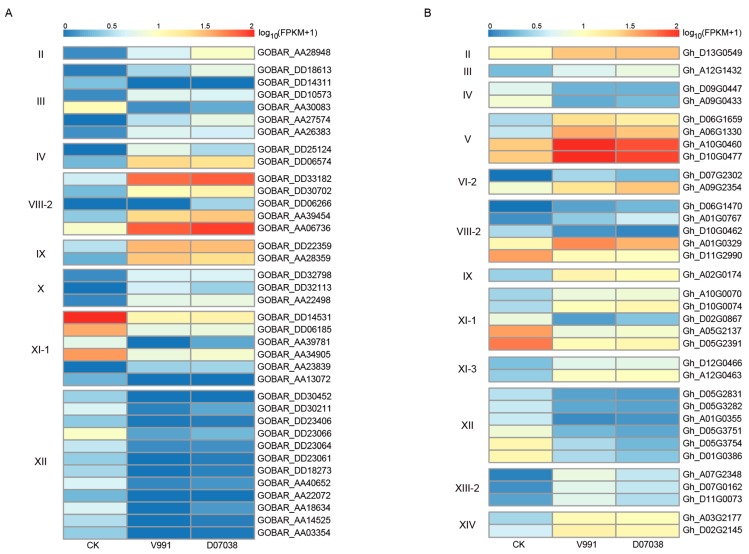
Expression patterns of differentially expressed *LRR-RLK* genes in response to *Verticillium dahlia* infection. For both *G. barbadense* (**A**) and *G. hirsutum* (**B**), significant differentially expressed *LRR-RLK* genes simultaneously detected in both samples infected with *V. dahliae* strain V991 (highly toxic) and samples infected with *V. dahliae* strain D07038 (intermediately toxic) were used for heatmap drawings for each subfamily.

Verticillium wilt is one of the most interactable diseases in cotton growth. The expression profiles of *Gossypium LRR-RLK* genes under Verticillium wilt infection were investigated to analyze their response to biotic stress. The main cultispecies *G. hirsutum* is susceptible to Verticillium wilt, while *G. barbadense* shows better resistance and immunity to Verticillium wilt. Transcriptomic dynamic response to Verticillium wilt was been studied between *G. hirsutum* and *G. barbadense* [59]. There were 37 significant differentially expressed (log_2_FC > 1 and FDR (false discovery rate) < 0.05) *LRR-RLK* genes detected in *G. barbadense* under two Verticillium wilt strain infections. The counterpart differentially expressed *LRR-RLK* gene numbers in *G. hirsutum* was 34. Among these differentially expressed *LRR-RLK* genes, subfamily XI, XII, and VIII-2 accounted for the largest share (Figure 8) in both *G. barbadense* and *G. hirsutum*. Comparing *G. barbadense* with *G. hirsutum*, we found that *G. barbadense* had more differentially expressed *LRR-RLK* genes belonging to subfamily XI-1 and XII than *G. hirsutum* (Figure 8). Expression heatmaps revealed that the majority of subfamily XI-1 and all subfamily XII *LRR-RLK* genes down-regulated significantly when infected by Verticillium wilt, while subfamily VIII-2 *LRR-RLK* genes were more likely to be up-regulated. Differentially expressed *LRR-RLK* gene belonging to subfamilies II, IX, V, VI, X, XI-3, XIII-2, and XIV were significantly up-regulated. The two homologous subfamily IV genes in *G. hirsutum* were significantly down-regulated. There was only one up-regulated subfamily III *LRR-RLK* gene which showed significant differential expression in *G. hirsutum*, while six subfamily III *LRR-RLK* genes, including two down-regulated and four up-regulated genes, were found to be differentially expressed in *G. barbadense*. The difference of LRR-RLK expression regulation between *G. barbadense* and *G. hirsutum* may be associated with a difference in disease resistance between these two species.

In summary, expression profiles of *LRR-RLK* genes varied both within and among subfamilies in *Gossypium* development and stress response, implying the functional divergence of *LRR-RLK* gene copies. It was difficult to assign distinct functional roles to different *Gossypium* LRR-RLK subfamilies, while the expression profile analysis in our study suggested wide involvement of *Gossypium LRR-RLK* genes in diverse processes of cotton development and stress response.

## 4. Conclusions

The present study performed a comprehensive analysis of the large *LRR-RLK* gene family in four *Gossypium* species. The *Gossypium LRR-RLK* genes were classified into 21 distinct subfamilies. Subfamilies XI and XII were found to be dramatically expanded in *Gossypium*, while tandem duplication was found to act as an important expansion mechanism in these expanded subfamilies. Functional and expression profile analysis revealed that *Gossypium LRR-RLK* genes were widely involved in diverse developmental processes and stress defenses. The expansion of subfamily XI and XII could be associated with more complicated development and regulation processes, and enhanced adaptability against various environments. The cis-acting regulatory elements analysis revealed that *Gossypium LRR-RLK* genes were extensively regulated by TFs and various abiotic and biotic stimuli. Our study provided valuable information for further functional study of *Gossypium LRR-RLK* genes.

## Figures and Tables

**Table 1:** Statistics of *A. thaliana* and *Gossypium LRR-RLK* gene distribution among different subfamilies. For both *A. thaliana* and four *Gossypium* species, the number of *LRR-RLK* genes belonging to each subfamily were counted respectively. The corresponding percentage in all *LRR-RLK* genes from specific species was computed and indicated in brackets.

Subfamily	Number (Percentage) of Genes in Each Subfamily				
	*A. thaliana*	*G. arboreum*	*G. barbadense*	*G. hirsutum*	*G. raimondii*
I	41 (19.2%)	4 (1.3%)	6 (1.2%)	7 (1.4%)	3 (0.9%)
II	14 (6.6%)	17 (5.7%)	23 (4.5%)	29 (5.6%)	18 (5.7%)
III	41 (19.2%)	45 (15.1%)	68 (13.3%)	83 (16.1%)	46 (14.5%)
IV	3 (1.4%)	5 (1.7%)	10 (2.0%)	10 (1.9%)	5 (1.6%)
V	9 (4.2%)	10 (3.4%)	15 (2.9%)	19 (3.7%)	9 (2.8%)
VI-1	5 (2.3%)	7 (2.3%)	11 (2.2%)	14 (2.7%)	7 (2.2%)
VI-2	4 (1.9%)	2 (0.7%)	2 (0.4%)	6 (1.2%)	3 (0.9%)
VII-1	2 (0.9%)	4 (1.3%)	11 (2.2%)	7 (1.4%)	4 (1.3%)
VII-2	5 (2.3%)	6 (2.0%)	6 (1.2%)	12 (2.3%)	6 (1.9%)
VIII-1	8 (3.8%)	4 (1.3%)	7 (1.4%)	7 (1.4%)	4 (1.3%)
VIII-2	12 (5.6%)	17 (5.7%)	26 (5.1%)	30 (5.8%)	17 (5.4%)
IX	4 (1.9%)	12 (4.0%)	18 (3.5%)	19 (3.7%)	11 (3.5%)
X	15 (7.0%)	18 (6.0%)	32 (6.3%)	35 (6.8%)	18 (5.7%)
XI-1	28 (13.1%)	75 (25.2%)	135 (26.4%)	132 (25.6%)	81 (25.6%)
XI-2	2 (0.9%)	3 (1.0%)	3 (0.6%)	7 (1.4%)	3 (0.9%)
XI-3	2 (0.9%)	1 (0.3%)	1 (0.2%)	2 (0.4%)	1 (0.3%)
XII	7 (3.3%)	49 (16.4%)	102 (20.0%)	61 (11.8%)	63 (19.9%)
XIII-1	3 (1.4%)	3 (1.0%)	6 (1.2%)	5 (1.0%)	2 (0.6%)
XIII-2	3 (1.4%)	3 (1.0%)	6 (1.2%)	6 (1.2%)	3 (0.9%)
XIV	2 (0.9%)	5 (1.7%)	10 (2.0%)	9 (1.7%)	5 (1.6%)
XV	3 (1.4%)	8 (2.7%)	13 (2.5%)	15 (2.9%)	8 (2.5%)

**Table 2:** Number of identified tandem duplicated *LRR-RLK* genes among different subfamilies in four *Gossypium* species. Number of tandem duplicated gene sets in each subfamily were indicated in brackets.

Subfamily	Number of Tandem Duplicated Genes (Gene Sets)			
	*G. arboreum*	*G. barbadense*	*G. hirsutum*	*G. raimondii*
II	5(2)	2(1)	5(2)	5(2)
III	2(1)	4(2)	4(2)	2(1)
IX	-	2(1)	-	-
VII-1		4(2)	-	-
VIII-2	10(4)	11(4)	14(6)	11(4)
XI-1	31(11)	59(19)	42(16)	35(9)
XI-2	-	-	2(1)	-
XII	31(8)	64(18)	25(6)	43(9)
Total	79(26)	146(47)	92(33)	96(25)

**Table 3:** Statistics of cis-acting regulatory elements detected in promoter regions of *Gossypium LRR-RLK* genes. (*cis-acting regulatory elements that have no functional description were not shown, see Table S3 for details.).

Element Species (ID of CARE)	Number (Percentage) of Elements in Promoters of *LRR-RLK* Genes				
	*A. thaliana*	*G. arboreum*	*G. barbadense*	*G. hirsutum*	*G. raimondii*
**core promoter/enhancer element** (AT-TATA-box, CAAT-box, TATA, TATA-box)	213(100.0%)	298(100.0%)	506(99.0%)	514(99.8%)	317(100.0%)
**water response** (AT-rich element, MYB)	206(96.7%)	286(96.0%)	469(91.8%)	492(95.5%)	293(92.4%)
**drought response** (ACTCATCCT sequence, as-1, DRE core, DRE1, MBS, MYB recognition site, MYC)	212(99.5%)	291(97.7%)	487(95.3%)	506(98.3%)	303(95.6%)
**cold response** (LTR)	2(0.9%)	-	-	-	3(0.9%)
**heat, osmotic stress, low pH, nutrient starvation stresses response** (STRE)	69(32.4%)	79(26.5%)	150(29.4%)	168(32.6%)	99(31.2%)
**anoxic response** (ARE, GC-motif)	89(41.8%)	202(67.8%)	337(65.9%)	347(67.4%)	181(57.1%)
**Cd response** (AP-1)	183(85.9%)	238(79.9%)	387(75.7%)	405(78.6%)	237(74.8%)
**defense response** (TC-rich repeats)	4(1.9%)	17(5.7%)	22(4.3%)	27(5.2%)	17(5.4%)
**wounding and pathogen response** (box S, W box, WRE3, WUN-motif)	89(41.8%)	110(36.9%)	167(32.7%)	181(35.1%)	111(35.0%)
**light response** (3-AF1 binding site, 4cl-CMA1b/1c/2b, AAAC-motif, ACA-motif, ACE, AE-box, AT1-motif, ATC-motif, ATCT-motif, Box 4, Box II, CAG-motif, chs-CMA1a/2a/2b/2c, chs-Unit 1 m1, GA-motif, Gap-box, GATA-motif, GATT-motif, G-Box, GGA-motif, GT1-motif, GTGGC-motif, I-box, LAMP-element, L-box, LS7, MRE, Pc-CMA2a, Pc-CMA2c, sbp-CMA1c, Sp1, TCCC-motif, TCT-motif)	166(77.9%)	241(80.9%)	407(79.6%)	425(82.5%)	255(80.4%)
**circadian response** (circadian)	213(100.0%)	298(100.0%)	503(98.4%)	512(99.4%)	314(99.1%)
**ETH response** (ERE)	150(70.4%)	160(53.7%)	268(52.4%)	299(58.1%)	182(57.4%)
**ABA response** (ABRE, ABRE2, ABRE3a, ABRE4, AT-ABRE, CARE)	105(49.3%)	87(29.2%)	142(27.8%)	137(26.6%)	104(32.8%)
**GA response** (GARE-motif, P-box, TATC-box, CARE)	94(44.1%)	226(75.8%)	354(69.3%)	370(71.8%)	248(78.2%)
**JA response** (CGTCA-motif, TGACG-motif, JERE)	14(6.6%)	19(6.4%)	39(7.6%)	40(7.8%)	22(6.9%)
**SA response** (SARE, TCA-element)	99(46.5%)	128(43.0%)	199(38.9%)	225(43.7%)	121(38.2%)
**auxin response** (TGA-element, TGA-box, AuxRR-core, AuxRE)	142(66.7%)	151(50.7%)	217(42.5%)	230(44.7%)	153(48.3%)
**cell cycle and cell proliferation response** (CCGTCC motif, dOCT, E2Fb, MSA-like, Myb-binding site, NON, OCT, re2f-1)	73(34.3%)	133(44.6%)	191(37.4%)	219(42.5%)	114(36.0%)
**tissue specific/preferential expressed** (AACA_motif, AC-I, AC-II, CAT-box, GCN4_motif, motif I, NON-box, RY-element, telo-box)	4(1.9%)	-	1(0.2%)	-	-

## Supplementary Materials

The following are available online at http://www.mdpi.com/2073-4425/9/12/592/s1. Figure S1: Domain and exon-intron organization of identified LRR-RLK family members in *A. thaliana* and *Gossypium*. Figure S2: NJ tree constructed by MEGA 7 based on amino acid sequences of LRR-RLKs from *A. thaliana* and *Gossypium*. Figure S3: Chromosomal location of *LRR-RLK* genes from four *Gossypium* species. Figure S4: GO enrichment results of Gossypium *LRR-RLK* genes. Table S1: Detailed assignment of *A. thaliana* and *Gossypium LRR-RLK* genes into different subfamilies. Table S2: Genomic distribution of Gossypium *LRR-RLK* genes among different chromosomes and scaffolds. Table S3: Number of tandem duplication gene sets and involved genes in *Gossypium* LRR-RLK family. Table S4: Statistics of cis-acting regulatory elements found by PlantCARE in promoter regions of *Gossypium LRR-RLK* genes. Table S5: Statistics of TF binding sites predicted in promoter regions of *Gossypium LRR-RLK* genes. Supplementary data 1: Multiple sequence alignment results of LRR-RLKs from *A. thaliana* and *Gossypium*. Supplementary data 2: Original ML tree constructed from amino acid sequences of LRR-RLKs from *A. thaliana* and *Gossypium*.
