# Supplementary material for: Genome-Wide Analysis of LRR-RLK Gene Family in Four Gossypium Species and Expression Analysis during Cotton Development and Stress Responses
Source: Genes (Basel). 2018 Nov 29;9(12):592. doi: 10.3390/genes9120592 (PMC6316826; doi:10.3390/genes9120592)
Supplement: Supplementary file 1 [file genes-09-00592-s001.zip › genes-373005-supplementary.docx]

**Figure S1: Domain and Exon-Intron Organization of Identified LRR-RLK Family Gene Members in *A. thaliana* and *Gossypium*.**

Exon-intron structures of all identified *LRR-RLK* genes from four *Gossypium* species and *A. thaliana*. LRR, KD, and TM domain coding regions were marked on exons by different colored rectangles. 21 subfamilies were distinguished by different color branches (see file Figure S1.pdf).

**Figure S2: NJ Tree Constructed by MEGA 7 Based on Amino Acid Sequences of LRR-RLKs from** ***A. thaliana* and *Gossypium*.**

Amino acid sequences of LRR-RLKs from *A. thaliana* and *Gossypium* were aligned by MUSCLE software with default parameters. Aligned sequences were used to constructed the NJ tree by MEGA 7 with 1000 bootstraps. Only topology of the tree was displayed. Bootstrap values lower than 50 were hidden (see file Figure S2.pdf).

**Figure S3: Chromosomal Location of *LRR-RLK* Genes From Four *Gossypium* Species.**

*LRR-RLK* genes from *G. arboreum*, *G. barbadense*, *G. hirsutum*, and *G. raimondii* were mapped to chromosomes based on chromosomal coordinates extracted from gene feature annotation files (shown by A, B, C, and D, respectively). *LRR-RLK* genes located on scaffolds were not shown. Tandem duplication gene sets were highlighted by red border rectangles (see file Figure S3.pdf).

**Figure S4: GO Enrichment Results of *Gossypium* *LRR-RLK* Genes.**

Results of *G. arboreum*, *G. barbadense*, *G. hirsutum,* and *G. raimondii* were shown by A, B, C, and D, respectively (see file Figure S4.pdf).

**Table S1: Detailed Assignment of *A. thaliana* and *Gossypium* *LRR-RLK* Genes Into Different Subfamilies.**

*LRR-RLK* genes from *A. thaliana* and *G. arboreum*, *G. barbadense*, *G. hirsutum*, and *G. raimondii* were assigned into 21 subfamilies.

**Table S2: Genomic Distribution of *Gossypium* *LRR-RLK* Genes Among Different Chromosomes and Scaffolds.**

For each *Gossypium* species, the number of *LRR-RLK* genes located on each chromosome and scaffold were counted. The corresponding percentages were indicated in brackets.

**Table S3: Number and Percentage of Tandem Duplication Genes in *Gossypium* LRR-RLK Family.**

For each LRR-RLK subfamily, the number of tandem duplication genes was counted. The percentage of tandem duplication genes out of all subfamily members was computed and indicated in brackets.

**Table S4: Statistics of *cis*-Acting Regulatory Elements Found by PlantCARE in Promoter Regions of *Gossypium* *LRR-RLK* Genes.**

Based on PlantCARE analysis results, 121 kinds of *cis*-acting regulatory elements were found in promoters of *Gossypium* *LRR-RLK* genes. Number of each kind of *cis*-acting element found in specific species was counted.

**Table S5: Statistics of TF Binding Sites Predicted in Promoter Regions of *Gossypium* *LRR-RLK* Genes.**

TF binding site analysis showed that *Gossypium* *LRR-RLK* genes could be regulated by TFs belonging to as many as 39 TF families. Number of *LRR-RLK* genes regulated by each TF family was counted for *G. arboreum*, *G. hirsutum,* and *G. raimondii*, respectively.

**Table S1**

| **Subfamily** | ***LRR-RLK* genes** | | | | |
| --- | --- | --- | --- | --- | --- |
|  | ***A. thaliana*** | ***G. arboreum*** | ***G. barbadense*** | ***G. hirsutum*** | ***G. raimondii*** |
| I | AT1G05700 | evm.model.Ga06G0711 | GOBAR_AA04072 | Gh_A06G0611 | Gorai.001G186800 |
|  | AT1G07550 | evm.model.Ga07G1767 | GOBAR_AA06016 | Gh_A07G1471 | Gorai.004G225900 |
|  | AT1G07560 | evm.model.Ga08G2286 | GOBAR_AA16884 | Gh_A08G1731 | Gorai.010G230800 |
|  | AT1G49100 | evm.model.Ga10G2407 | GOBAR_DD09002 | Gh_A10G0566 |  |
|  | AT1G51790 |  | GOBAR_DD35797 | Gh_D07G1567 |  |
|  | AT1G51800 |  | GOBAR_DD37501 | Gh_D08G2082 |  |
|  | AT1G51820 |  |  | Gh_D10G0606 |  |
|  | AT1G51830 |  |  |  |  |
|  | AT1G51850 |  |  |  |  |
|  | AT1G51860 |  |  |  |  |
|  | AT1G51870 |  |  |  |  |
|  | AT1G51880 |  |  |  |  |
|  | AT1G51890 |  |  |  |  |
|  | AT1G51910 |  |  |  |  |
|  | AT1G67720 |  |  |  |  |
|  | AT2G04300 |  |  |  |  |
|  | AT2G14440 |  |  |  |  |
|  | AT2G14510 |  |  |  |  |
|  | AT2G19190 |  |  |  |  |
|  | AT2G19210 |  |  |  |  |
|  | AT2G19230 |  |  |  |  |
|  | AT2G28960 |  |  |  |  |
|  | AT2G28970 |  |  |  |  |
|  | AT2G28990 |  |  |  |  |
|  | AT2G37050 |  |  |  |  |
|  | AT3G21340 |  |  |  |  |
|  | AT3G46330 |  |  |  |  |
|  | AT3G46340 |  |  |  |  |
|  | AT3G46350 |  |  |  |  |
|  | AT3G46370 |  |  |  |  |
|  | AT3G46400 |  |  |  |  |
|  | AT3G46420 |  |  |  |  |
|  | AT4G20450 |  |  |  |  |
|  | AT4G29180 |  |  |  |  |
|  | AT4G29450 |  |  |  |  |
|  | AT4G29990 |  |  |  |  |
|  | AT5G16900 |  |  |  |  |
|  | AT5G48740 |  |  |  |  |
|  | AT5G59650 |  |  |  |  |
|  | AT5G59670 |  |  |  |  |
|  | AT5G59680 |  |  |  |  |
| II | AT5G16000 | evm.model.Ga02G0374 | GOBAR_AA06626 | Gh_A02G1363 | Gorai.001G241500 |
|  | AT1G34210 | evm.model.Ga03G2276 | GOBAR_AA13618 | Gh_A04G0958 | Gorai.003G039900 |
|  | AT1G60800 | evm.model.Ga04G0167 | GOBAR_AA17832 | Gh_A05G2653 | Gorai.005G218200 |
|  | AT1G71830 | evm.model.Ga04G0168 | GOBAR_AA22524 | Gh_A05G3984 | Gorai.007G060500 |
|  | AT2G13790 | evm.model.Ga04G1123 | GOBAR_AA26118 | Gh_A07G1895 | Gorai.007G186300 |
|  | AT2G13800 | evm.model.Ga05G0081 | GOBAR_AA27404 | Gh_A10G0056 | Gorai.007G236500 |
|  | AT2G23950 | evm.model.Ga05G3363 | GOBAR_AA28948 | Gh_A10G0332 | Gorai.009G008400 |
|  | AT3G25560 | evm.model.Ga07G2411 | GOBAR_AA33225 | Gh_A11G0480 | Gorai.009G327400 |
|  | AT4G30520 | evm.model.Ga10G2706 | GOBAR_AA38005 | Gh_A11G1548 | Gorai.009G337000 |
|  | AT4G33430 | evm.model.Ga10G3033 | GOBAR_AA40607 | Gh_A11G1848 | Gorai.011G006900 |
|  | AT5G10290 | evm.model.Ga11G1655 | GOBAR_DD00372 | Gh_A13G0791 | Gorai.011G038400 |
|  | AT5G45780 | evm.model.Ga11G2239 | GOBAR_DD01050 | Gh_D02G1987 | Gorai.012G097800 |
|  | AT5G63710 | evm.model.Ga11G3528 | GOBAR_DD05841 | Gh_D04G0838 | Gorai.012G140900 |
|  | AT5G65240 | evm.model.Ga13G0575 | GOBAR_DD09365 | Gh_D04G1502 | Gorai.012G141000 |
|  |  | evm.model.Ga13G0576 | GOBAR_DD14343 | Gh_D04G1503 | Gorai.013G063000 |
|  |  | evm.model.Ga13G0579 | GOBAR_DD20162 | Gh_D05G0066 | Gorai.013G063100 |
|  |  | evm.model.Ga13G1206 | GOBAR_DD24671 | Gh_D05G3027 | Gorai.013G063200 |
|  |  |  | GOBAR_DD27391 | Gh_D05G3785 | Gorai.013G102600 |
|  |  |  | GOBAR_DD28293 | Gh_D07G2110 |  |
|  |  |  | GOBAR_DD28384 | Gh_D10G0063 |  |
|  |  |  | GOBAR_DD33945 | Gh_D10G0338 |  |
|  |  |  | GOBAR_DD34459 | Gh_D11G0558 |  |
|  |  |  | GOBAR_DD35041 | Gh_D11G2163 |  |
|  |  |  |  | Gh_D11G3483 |  |
|  |  |  |  | Gh_D13G0549 |  |
|  |  |  |  | Gh_D13G0550 |  |
|  |  |  |  | Gh_D13G0551 |  |
|  |  |  |  | Gh_D13G0931 |  |
|  |  |  |  | Gh_Sca007506G01 |  |
| III | AT1G25320 | evm.model.Ga01G0927 | GOBAR_AA01897 | Gh_A01G0679 | Gorai.001G016300 |
|  | AT1G48480 | evm.model.Ga01G1414 | GOBAR_AA02586 | Gh_A01G1662 | Gorai.001G070200 |
|  | AT1G50610 | evm.model.Ga01G1734 | GOBAR_AA04295 | Gh_A01G1797 | Gorai.001G081900 |
|  | AT1G60630 | evm.model.Ga01G2462 | GOBAR_AA04771 | Gh_A02G1286 | Gorai.001G198100 |
|  | AT1G64210 | evm.model.Ga02G0236 | GOBAR_AA05833 | Gh_A02G1481 | Gorai.001G203900 |
|  | AT1G66830 | evm.model.Ga02G0246 | GOBAR_AA09778 | Gh_A02G1491 | Gorai.002G096000 |
|  | AT1G67510 | evm.model.Ga02G0440 | GOBAR_AA09931 | Gh_A03G0187 | Gorai.002G134400 |
|  | AT1G68400 | evm.model.Ga02G1450 | GOBAR_AA12272 | Gh_A03G0740 | Gorai.002G229000 |
|  | AT2G01210 | evm.model.Ga02G1610 | GOBAR_AA12282 | Gh_A03G1108 | Gorai.002G243600 |
|  | AT2G07040 | evm.model.Ga03G1241 | GOBAR_AA12723 | Gh_A03G1313 | Gorai.003G026300 |
|  | AT2G15300 | evm.model.Ga03G1706 | GOBAR_AA13939 | Gh_A05G0010 | Gorai.003G046400 |
|  | AT2G23300 | evm.model.Ga03G2001 | GOBAR_AA14051 | Gh_A05G0258 | Gorai.003G082600 |
|  | AT2G26730 | evm.model.Ga05G0001 | GOBAR_AA14423 | Gh_A05G0869 | Gorai.003G153000 |
|  | AT2G27060 | evm.model.Ga05G0368 | GOBAR_AA14561 | Gh_A05G0985 | Gorai.004G024700 |
|  | AT2G36570 | evm.model.Ga05G1069 | GOBAR_AA14974 | Gh_A05G1156 | Gorai.005G120900 |
|  | AT2G42290 | evm.model.Ga05G1218 | GOBAR_AA15515 | Gh_A05G1773 | Gorai.005G169100 |
|  | AT3G02880 | evm.model.Ga05G1478 | GOBAR_AA17265 | Gh_A06G1704 | Gorai.005G192800 |
|  | AT3G08680 | evm.model.Ga05G2179 | GOBAR_AA18336 | Gh_A06G1972 | Gorai.005G193500 |
|  | AT3G17840 | evm.model.Ga06G1337 | GOBAR_AA19571 | Gh_A06G2116 | Gorai.007G152400 |
|  | AT3G20190 | evm.model.Ga06G1967 | GOBAR_AA20033 | Gh_A07G0025 | Gorai.007G184800 |
|  | AT3G24660 | evm.model.Ga06G2494 | GOBAR_AA20692 | Gh_A07G1510 | Gorai.007G186000 |
|  | AT3G42880 | evm.model.Ga07G0032 | GOBAR_AA22372 | Gh_A07G1590 | Gorai.007G216500 |
|  | AT3G50230 | evm.model.Ga07G0171 | GOBAR_AA22997 | Gh_A07G2365 | Gorai.007G244000 |
|  | AT3G51740 | evm.model.Ga07G0850 | GOBAR_AA24237 | Gh_A08G2132 | Gorai.008G023200 |
|  | AT3G56100 | evm.model.Ga07G1977 | GOBAR_AA26383 | Gh_A10G0041 | Gorai.008G078600 |
|  | AT3G57830 | evm.model.Ga07G2006 | GOBAR_AA27574 | Gh_A10G1605 | Gorai.008G171200 |
|  | AT4G23740 | evm.model.Ga08G0254 | GOBAR_AA28465 | Gh_A10G2139 | Gorai.008G271600 |
|  | AT4G31250 | evm.model.Ga08G2823 | GOBAR_AA29546 | Gh_A10G2243 | Gorai.009G001100 |
|  | AT4G34220 | evm.model.Ga10G0067 | GOBAR_AA30083 | Gh_A11G1535 | Gorai.009G036600 |
|  | AT4G37250 | evm.model.Ga10G0930 | GOBAR_AA31571 | Gh_A11G1546 | Gorai.009G105200 |
|  | AT5G05160 | evm.model.Ga10G2485 | GOBAR_AA33222 | Gh_A11G1799 | Gorai.009G120000 |
|  | AT5G10020 | evm.model.Ga10G3051 | GOBAR_AA35240 | Gh_A11G1821 | Gorai.009G214700 |
|  | AT5G16590 | evm.model.Ga10G3080 | GOBAR_AA35512 | Gh_A11G3081 | Gorai.010G067400 |
|  | AT5G20690 | evm.model.Ga11G1573 | GOBAR_AA40789 | Gh_A12G0196 | Gorai.010G135900 |
|  | AT5G24100 | evm.model.Ga11G1909 | GOBAR_DD00395 | Gh_A12G1366 | Gorai.010G194100 |
|  | AT5G35390 | evm.model.Ga11G2241 | GOBAR_DD02308 | Gh_A12G1432 | Gorai.010G235300 |
|  | AT5G43020 | evm.model.Ga11G2253 | GOBAR_DD03453 | Gh_A12G2709 | Gorai.011G001900 |
|  | AT5G53320 | evm.model.Ga11G2612 | GOBAR_DD03791 | Gh_A13G0381 | Gorai.011G004900 |
|  | AT5G58300 | evm.model.Ga12G0269 | GOBAR_DD09415 | Gh_A13G1140 | Gorai.011G061900 |
|  | AT5G67200 | evm.model.Ga12G1290 | GOBAR_DD10573 | Gh_A13G1753 | Gorai.011G206900 |
|  | AT5G67280 | evm.model.Ga12G1371 | GOBAR_DD10653 | Gh_D01G0700 | Gorai.011G209000 |
|  |  | evm.model.Ga13G0423 | GOBAR_DD11401 | Gh_D01G1048 | Gorai.011G289900 |
|  |  | evm.model.Ga13G1701 | GOBAR_DD12338 | Gh_D01G1911 | Gorai.012G184100 |
|  |  | evm.model.Ga13G2444 | GOBAR_DD12763 | Gh_D01G2038 | Gorai.013G047000 |
|  |  | evm.model.Ga14G0146 | GOBAR_DD13016 | Gh_D02G1532 | Gorai.013G156100 |
|  |  |  | GOBAR_DD13965 | Gh_D02G1751 | Gorai.013G231800 |
|  |  |  | GOBAR_DD14311 | Gh_D03G0223 |  |
|  |  |  | GOBAR_DD16051 | Gh_D03G0233 |  |
|  |  |  | GOBAR_DD17143 | Gh_D03G0426 |  |
|  |  |  | GOBAR_DD18442 | Gh_D03G0717 |  |
|  |  |  | GOBAR_DD18613 | Gh_D03G1395 |  |
|  |  |  | GOBAR_DD19748 | Gh_D04G1877 |  |
|  |  |  | GOBAR_DD20013 | Gh_D05G0014 |  |
|  |  |  | GOBAR_DD20067 | Gh_D05G0350 |  |
|  |  |  | GOBAR_DD22450 | Gh_D05G1096 |  |
|  |  |  | GOBAR_DD23035 | Gh_D05G1333 |  |
|  |  |  | GOBAR_DD23995 | Gh_D05G1967 |  |
|  |  |  | GOBAR_DD25022 | Gh_D05G3881 |  |
|  |  |  | GOBAR_DD26124 | Gh_D06G0562 |  |
|  |  |  | GOBAR_DD28760 | Gh_D06G1236 |  |
|  |  |  | GOBAR_DD29294 | Gh_D06G1758 |  |
|  |  |  | GOBAR_DD30362 | Gh_D07G0033 |  |
|  |  |  | GOBAR_DD31581 | Gh_D07G0618 |  |
|  |  |  | GOBAR_DD33021 | Gh_D07G1779 |  |
|  |  |  | GOBAR_DD34844 | Gh_D08G0214 |  |
|  |  |  | GOBAR_DD35427 | Gh_D08G2501 |  |
|  |  |  | GOBAR_DD35666 | Gh_D09G1616 |  |
|  |  |  | GOBAR_DD38225 | Gh_D10G0014 |  |
|  |  |  |  | Gh_D10G0044 |  |
|  |  |  |  | Gh_D10G0530 |  |
|  |  |  |  | Gh_D10G1114 |  |
|  |  |  |  | Gh_D10G1860 |  |
|  |  |  |  | Gh_D10G2460 |  |
|  |  |  |  | Gh_D11G1399 |  |
|  |  |  |  | Gh_D11G1700 |  |
|  |  |  |  | Gh_D11G1961 |  |
|  |  |  |  | Gh_D11G3486 |  |
|  |  |  |  | Gh_D12G0198 |  |
|  |  |  |  | Gh_D12G1552 |  |
|  |  |  |  | Gh_D12G2665 |  |
|  |  |  |  | Gh_D13G0424 |  |
|  |  |  |  | Gh_D13G1423 |  |
|  |  |  |  | Gh_D13G2101 |  |
| IV | AT2G45340 | evm.model.Ga08G1612 | GOBAR_AA03974 | Gh_A08G1211 | Gorai.004G162200 |
|  | AT4G22730 | evm.model.Ga08G2049 | GOBAR_AA07839 | Gh_A08G1564 | Gorai.004G202400 |
|  | AT5G51560 | evm.model.Ga09G0608 | GOBAR_AA11854 | Gh_A09G0433 | Gorai.006G064800 |
|  |  | evm.model.Ga12G0142 | GOBAR_AA19551 | Gh_A12G2019 | Gorai.008G239500 |
|  |  | evm.model.Ga12G0579 | GOBAR_AA30036 | Gh_A12G2393 | Gorai.008G284000 |
|  |  |  | GOBAR_DD02908 | Gh_D08G1494 |  |
|  |  |  | GOBAR_DD03499 | Gh_D08G1871 |  |
|  |  |  | GOBAR_DD11217 | Gh_D09G0447 |  |
|  |  |  | GOBAR_DD14824 | Gh_D12G2197 |  |
|  |  |  | GOBAR_DD26203 | Gh_D12G2520 |  |
| V | AT1G11130 | evm.model.Ga03G2114 | GOBAR_AA12628 | Gh_A03G1397 | Gorai.005G204500 |
|  | AT1G53730 | evm.model.Ga05G1223 | GOBAR_AA15761 | Gh_A05G1186 | Gorai.006G141800 |
|  | AT1G78980 | evm.model.Ga05G1509 | GOBAR_AA25683 | Gh_A06G1240 | Gorai.009G120500 |
|  | AT2G20850 | evm.model.Ga06G1334 | GOBAR_AA26005 | Gh_A06G1330 | Gorai.009G148500 |
|  | AT3G13065 | evm.model.Ga06G1732 | GOBAR_AA31899 | Gh_A09G1189 | Gorai.010G135500 |
|  | AT3G14350 | evm.model.Ga06G1844 | GOBAR_AA39764 | Gh_A10G0105 | Gorai.010G172500 |
|  | AT4G03390 | evm.model.Ga09G1450 | GOBAR_DD02568 | Gh_A10G0221 | Gorai.010G183400 |
|  | AT4G22130 | evm.model.Ga10G2556 | GOBAR_DD03326 | Gh_A10G0460 | Gorai.011G011800 |
|  | AT5G06820 | evm.model.Ga10G2845 | GOBAR_DD06574 | Gh_D02G1857 | Gorai.011G024500 |
|  |  | evm.model.Ga10G2982 | GOBAR_DD13117 | Gh_D05G1102 |  |
|  |  |  | GOBAR_DD16752 | Gh_D05G1364 |  |
|  |  |  | GOBAR_DD17332 | Gh_D06G1230 |  |
|  |  |  | GOBAR_DD23975 | Gh_D06G1559 |  |
|  |  |  | GOBAR_DD25124 | Gh_D06G1659 |  |
|  |  |  | GOBAR_DD30456 | Gh_D09G1195 |  |
|  |  |  |  | Gh_D10G0109 |  |
|  |  |  |  | Gh_D10G0200 |  |
|  |  |  |  | Gh_D10G0477 |  |
|  |  |  |  | Gh_Sca005242G02 |  |
| VI-1 | AT1G14390 | evm.model.Ga04G1215 | GOBAR_AA16586 | Gh_A05G2964 | Gorai.001G060100 |
|  | AT2G02780 | evm.model.Ga06G2091 | GOBAR_AA17502 | Gh_A06G1500 | Gorai.001G269200 |
|  | AT3G03770 | evm.model.Ga07G0618 | GOBAR_AA17728 | Gh_A07G0463 | Gorai.007G256600 |
|  | AT5G14210 | evm.model.Ga07G2628 | GOBAR_AA21815 | Gh_A07G2084 | Gorai.008G205300 |
|  | AT5G63410 | evm.model.Ga11G1374 | GOBAR_AA33642 | Gh_A11G2069 | Gorai.012G088400 |
|  |  | evm.model.Ga12G0942 | GOBAR_DD01474 | Gh_A12G1707 | Gorai.013G195600 |
|  |  | evm.model.Ga13G2034 | GOBAR_DD07211 | Gh_A13G1410 | Gorai.N006900 |
|  |  |  | GOBAR_DD15827 | Gh_D04G0754 |  |
|  |  |  | GOBAR_DD33655 | Gh_D06G1866 |  |
|  |  |  | GOBAR_DD35972 | Gh_D07G0527 |  |
|  |  |  | GOBAR_DD36971 | Gh_D07G2302 |  |
|  |  |  |  | Gh_D11G2375 |  |
|  |  |  |  | Gh_D12G1869 |  |
|  |  |  |  | Gh_D13G1725 |  |
| VI-2 | AT1G63430 | evm.model.Ga06G1985 | GOBAR_AA08392 | Gh_A06G1427 | Gorai.006G209800 |
|  | AT4G18640 | evm.model.Ga09G2157 | GOBAR_DD24916 | Gh_A06G2018 | Gorai.010G195700 |
|  | AT5G41180 |  |  | Gh_A09G2354 | Gorai.010G241800 |
|  | AT5G45840 |  |  | Gh_D06G1775 |  |
|  |  |  |  | Gh_D06G2144 |  |
|  |  |  |  | Gh_D09G1830 |  |
| VII-1 | AT1G75640 | evm.model.Ga02G0107 | GOBAR_AA05718 | Gh_A02G1622 | Gorai.003G011000 |
|  | AT4G36180 | evm.model.Ga05G2732 | GOBAR_AA12818 | Gh_A05G2190 | Gorai.007G196700 |
|  |  | evm.model.Ga06G0064 | GOBAR_AA12819 | Gh_A06G1833 | Gorai.009G271000 |
|  |  | evm.model.Ga11G2119 | GOBAR_AA15481 | Gh_A11G1638 | Gorai.010G006200 |
|  |  |  | GOBAR_AA23346 | Gh_D05G2451 |  |
|  |  |  | GOBAR_AA31412 | Gh_D06G0018 |  |
|  |  |  | GOBAR_DD05180 | Gh_D11G1796 |  |
|  |  |  | GOBAR_DD14192 |  |  |
|  |  |  | GOBAR_DD21077 |  |  |
|  |  |  | GOBAR_DD27229 |  |  |
|  |  |  | GOBAR_DD37192 |  |  |
| VII-2 | AT1G12460 | evm.model.Ga06G2187 | GOBAR_AA03957 | Gh_A06G1577 | Gorai.004G204100 |
|  | AT1G62950 | evm.model.Ga08G2066 | GOBAR_AA18887 | Gh_A08G1580 | Gorai.006G227000 |
|  | AT3G28040 | evm.model.Ga09G2340 | GOBAR_AA33808 | Gh_A09G1878 | Gorai.008G241300 |
|  | AT3G56370 | evm.model.Ga10G0920 | GOBAR_DD00413 | Gh_A10G1339 | Gorai.010G218400 |
|  | AT5G01890 | evm.model.Ga10G1819 | GOBAR_DD11366 | Gh_A10G1611 | Gorai.011G127000 |
|  |  | evm.model.Ga12G0561 | GOBAR_DD22294 | Gh_A12G2037 | Gorai.011G209800 |
|  |  |  |  | Gh_D06G1931 |  |
|  |  |  |  | Gh_D08G1887 |  |
|  |  |  |  | Gh_D09G1983 |  |
|  |  |  |  | Gh_D10G1132 |  |
|  |  |  |  | Gh_D10G1867 |  |
|  |  |  |  | Gh_D12G2215 |  |
| VIII-1 | AT1G06840 | evm.model.Ga03G0019 | GOBAR_AA00944 | Gh_A02G0017 | Gorai.005G002700 |
|  | AT1G79620 | evm.model.Ga03G0992 | GOBAR_AA01222 | Gh_A02G0899 | Gorai.005G121200 |
|  | AT3G53590 | evm.model.Ga10G1839 | GOBAR_DD08744 | Gh_A06G1925 | Gorai.010G221900 |
|  | AT5G01950 | evm.model.Ga13G2840 | GOBAR_DD12473 | Gh_A13G2056 | Gorai.013G271600 |
|  | AT5G37450 |  | GOBAR_DD12898 | Gh_D02G1064 |  |
|  | AT5G49760 |  | GOBAR_DD34553 | Gh_D06G1961 |  |
|  | AT5G49770 |  | GOBAR_DD37404 | Gh_D13G2457 |  |
|  | AT5G49780 |  |  |  |  |
| VIII-2 | AT1G07650 | evm.model.Ga01G0485 | GOBAR_AA06728 | Gh_A01G0326 | Gorai.001G131900 |
|  | AT1G29720 | evm.model.Ga01G0487 | GOBAR_AA06736 | Gh_A01G0328 | Gorai.002G048600 |
|  | AT1G29730 | evm.model.Ga01G0488 | GOBAR_AA09541 | Gh_A01G0329 | Gorai.002G048900 |
|  | AT1G29740 | evm.model.Ga01G1035 | GOBAR_AA10846 | Gh_A01G0767 | Gorai.002G049000 |
|  | AT1G29750 | evm.model.Ga05G1621 | GOBAR_AA15268 | Gh_A04G0369 | Gorai.002G105800 |
|  | AT1G53420 | evm.model.Ga05G1622 | GOBAR_AA16893 | Gh_A05G1284 | Gorai.006G003800 |
|  | AT1G53430 | evm.model.Ga05G1623 | GOBAR_AA18203 | Gh_A09G0031 | Gorai.007G330300 |
|  | AT1G53440 | evm.model.Ga05G3732 | GOBAR_AA20099 | Gh_A09G0731 | Gorai.007G330600 |
|  | AT1G56120 | evm.model.Ga06G1627 | GOBAR_AA27911 | Gh_A10G0444 | Gorai.007G330700 |
|  | AT1G56130 | evm.model.Ga09G0037 | GOBAR_AA39454 | Gh_A10G0445 | Gorai.009G159400 |
|  | AT1G56140 | evm.model.Ga09G0920 | GOBAR_DD03303 | Gh_A11G2604 | Gorai.009G159500 |
|  | AT3G14840 | evm.model.Ga10G2575 | GOBAR_DD04145 | Gh_D01G0368 | Gorai.009G363600 |
|  |  | evm.model.Ga10G2576 | GOBAR_DD06266 | Gh_D01G0369 | Gorai.010G163000 |
|  |  | evm.model.Ga11G0666 | GOBAR_DD08094 | Gh_D01G0370 | Gorai.011G052100 |
|  |  | evm.model.Ga11G0669 | GOBAR_DD23071 | Gh_D01G0787 | Gorai.011G052300 |
|  |  | evm.model.Ga11G1347 | GOBAR_DD23653 | Gh_D05G1456 | Gorai.011G052400 |
|  |  | evm.model.Ga14G1986 | GOBAR_DD28249 | Gh_D05G1457 | Gorai.N011200 |
|  |  |  | GOBAR_DD29676 | Gh_D05G3270 |  |
|  |  |  | GOBAR_DD30699 | Gh_D05G3835 |  |
|  |  |  | GOBAR_DD30700 | Gh_D06G1470 |  |
|  |  |  | GOBAR_DD30702 | Gh_D07G1170 |  |
|  |  |  | GOBAR_DD31635 | Gh_D09G0029 |  |
|  |  |  | GOBAR_DD32829 | Gh_D09G0732 |  |
|  |  |  | GOBAR_DD33182 | Gh_D10G0461 |  |
|  |  |  | GOBAR_DD35293 | Gh_D10G0462 |  |
|  |  |  | GOBAR_DD35294 | Gh_D11G2990 |  |
|  |  |  |  | Gh_D11G2994 |  |
|  |  |  |  | Gh_D11G3392 |  |
|  |  |  |  | Gh_Sca004803G06 |  |
|  |  |  |  | Gh_Sca008374G01 |  |
| IX | AT1G24650 | evm.model.Ga02G1518 | GOBAR_AA00579 | Gh_A01G1719 | Gorai.001G125200 |
|  | AT1G66150 | evm.model.Ga03G0219 | GOBAR_AA01355 | Gh_A02G0174 | Gorai.002G235300 |
|  | AT2G01820 | evm.model.Ga06G1442 | GOBAR_AA08174 | Gh_A07G1031 | Gorai.005G026700 |
|  | AT3G23750 | evm.model.Ga07G1227 | GOBAR_AA11520 | Gh_A09G0936 | Gorai.006G115200 |
|  |  | evm.model.Ga09G1161 | GOBAR_AA11645 | Gh_A09G1266 | Gorai.006G149500 |
|  |  | evm.model.Ga09G1532 | GOBAR_AA21683 | Gh_A10G1150 | Gorai.006G224800 |
|  |  | evm.model.Ga09G2315 | GOBAR_AA28359 | Gh_A11G1558 | Gorai.007G187900 |
|  |  | evm.model.Ga10G1550 | GOBAR_AA30589 | Gh_A12G1422 | Gorai.008G170100 |
|  |  | evm.model.Ga11G2222 | GOBAR_AA33268 | Gh_A13G0257 | Gorai.010G147700 |
|  |  | evm.model.Ga12G1303 | GOBAR_DD00463 | Gh_D01G1969 | Gorai.011G151900 |
|  |  | evm.model.Ga13G0306 | GOBAR_DD02534 | Gh_D02G0238 | Gorai.013G030200 |
|  |  | evm.model.Ga14G2553 | GOBAR_DD02535 | Gh_D07G1110 |  |
|  |  |  | GOBAR_DD21842 | Gh_D09G0963 |  |
|  |  |  | GOBAR_DD22359 | Gh_D09G1268 |  |
|  |  |  | GOBAR_DD26514 | Gh_D09G1973 |  |
|  |  |  | GOBAR_DD26741 | Gh_D10G1348 |  |
|  |  |  | GOBAR_DD28668 | Gh_D11G1715 |  |
|  |  |  | GOBAR_DD34146 | Gh_D12G1541 |  |
|  |  |  |  | Gh_D13G0274 |  |
| X | AT1G27190 | evm.model.Ga01G0643 | GOBAR_AA00789 | Gh_A01G0449 | Gorai.001G032100 |
|  | AT1G55610 | evm.model.Ga01G1648 | GOBAR_AA11783 | Gh_A02G1305 | Gorai.001G226700 |
|  | AT1G69990 | evm.model.Ga02G0457 | GOBAR_AA16459 | Gh_A03G0965 | Gorai.002G068500 |
|  | AT1G72300 | evm.model.Ga02G0752 | GOBAR_AA17207 | Gh_A04G0005 | Gorai.002G149600 |
|  | AT2G01950 | evm.model.Ga03G1508 | GOBAR_AA18512 | Gh_A04G0283 | Gorai.002G171000 |
|  | AT2G02220 | evm.model.Ga05G0565 | GOBAR_AA22498 | Gh_A05G0422 | Gorai.003G048200 |
|  | AT2G24230 | evm.model.Ga05G3892 | GOBAR_AA22717 | Gh_A06G0877 | Gorai.004G120000 |
|  | AT3G13380 | evm.model.Ga06G1098 | GOBAR_AA24479 | Gh_A06G1161 | Gorai.005G151100 |
|  | AT3G28450 | evm.model.Ga06G1601 | GOBAR_AA25396 | Gh_A08G0893 | Gorai.007G038800 |
|  | AT4G39400 | evm.model.Ga07G0328 | GOBAR_AA27368 | Gh_A09G1610 | Gorai.007G062800 |
|  | AT5G07280 | evm.model.Ga08G1200 | GOBAR_AA29046 | Gh_A10G0005 | Gorai.008G195900 |
|  | AT5G45800 | evm.model.Ga09G2017 | GOBAR_AA31550 | Gh_A11G0297 | Gorai.009G055800 |
|  | AT5G48380 | evm.model.Ga10G3087 | GOBAR_AA33659 | Gh_A11G0504 | Gorai.009G381200 |
|  | AT5G53890 | evm.model.Ga11G3505 | GOBAR_AA35519 | Gh_A11G3159 | Gorai.009G455100 |
|  | AT5G58150 | evm.model.Ga11G3741 | GOBAR_AA37869 | Gh_A12G1634 | Gorai.010G112800 |
|  |  | evm.model.Ga12G1030 | GOBAR_AA39092 | Gh_A13G1618 | Gorai.010G160000 |
|  |  | evm.model.Ga13G2297 | GOBAR_DD00844 | Gh_D01G0459 | Gorai.011G001200 |
|  |  | evm.model.Ga14G0646 | GOBAR_DD03950 | Gh_D01G1333 | Gorai.013G216200 |
|  |  |  | GOBAR_DD05380 | Gh_D02G1350 |  |
|  |  |  | GOBAR_DD06389 | Gh_D03G0445 |  |
|  |  |  | GOBAR_DD07206 | Gh_D05G0540 |  |
|  |  |  | GOBAR_DD10547 | Gh_D05G3382 |  |
|  |  |  | GOBAR_DD14819 | Gh_D05G3727 |  |
|  |  |  | GOBAR_DD18646 | Gh_D06G1027 |  |
|  |  |  | GOBAR_DD22119 | Gh_D06G1442 |  |
|  |  |  | GOBAR_DD22799 | Gh_D07G0266 |  |
|  |  |  | GOBAR_DD26786 | Gh_D08G1085 |  |
|  |  |  | GOBAR_DD32113 | Gh_D10G0007 |  |
|  |  |  | GOBAR_DD32438 | Gh_D11G0354 |  |
|  |  |  | GOBAR_DD32798 | Gh_D11G0582 |  |
|  |  |  | GOBAR_DD33160 | Gh_D11G0725 |  |
|  |  |  | GOBAR_DD38060 | Gh_D11G2243 |  |
|  |  |  |  | Gh_D12G1777 |  |
|  |  |  |  | Gh_D13G1978 |  |
|  |  |  |  | Gh_Sca004882G01 |  |
| XI-1 | AT1G08590 | evm.model.Ga01G1002 | GOBAR_AA03496 | Gh_A01G0744 | Gorai.001G026000 |
|  | AT1G09970 | evm.model.Ga02G0316 | GOBAR_AA03653 | Gh_A01G1507 | Gorai.001G026700 |
|  | AT1G17230 | evm.model.Ga02G0318 | GOBAR_AA06609 | Gh_A01G1549 | Gorai.001G049500 |
|  | AT1G17750 | evm.model.Ga02G1315 | GOBAR_AA07944 | Gh_A02G0270 | Gorai.001G180300 |
|  | AT1G28440 | evm.model.Ga03G0339 | GOBAR_AA07945 | Gh_A02G0428 | Gorai.001G181900 |
|  | AT1G34110 | evm.model.Ga03G0572 | GOBAR_AA07946 | Gh_A02G0677 | Gorai.001G184400 |
|  | AT1G35710 | evm.model.Ga03G0773 | GOBAR_AA07948 | Gh_A02G0816 | Gorai.001G237400 |
|  | AT1G72180 | evm.model.Ga03G1039 | GOBAR_AA08308 | Gh_A02G0817 | Gorai.002G103300 |
|  | AT1G73080 | evm.model.Ga03G1252 | GOBAR_AA08311 | Gh_A02G0853 | Gorai.002G211900 |
|  | AT1G75820 | evm.model.Ga03G2074 | GOBAR_AA08312 | Gh_A02G1416 | Gorai.002G217000 |
|  | AT2G33170 | evm.model.Ga03G2763 | GOBAR_AA08625 | Gh_A02G1417 | Gorai.003G034200 |
|  | AT3G19700 | evm.model.Ga03G2793 | GOBAR_AA09505 | Gh_A02G1419 | Gorai.003G034400 |
|  | AT3G24240 | evm.model.Ga05G0275 | GOBAR_AA10737 | Gh_A03G0775 | Gorai.003G034500 |
|  | AT3G49670 | evm.model.Ga05G0486 | GOBAR_AA10985 | Gh_A03G1368 | Gorai.004G124300 |
|  | AT4G08850 | evm.model.Ga05G0496 | GOBAR_AA11141 | Gh_A03G1912 | Gorai.004G222100 |
|  | AT4G20270 | evm.model.Ga05G0537 | GOBAR_AA11881 | Gh_A03G1939 | Gorai.004G249900 |
|  | AT4G26540 | evm.model.Ga05G0573 | GOBAR_AA11882 | Gh_A04G0038 | Gorai.005G039600 |
|  | AT4G28490 | evm.model.Ga05G0575 | GOBAR_AA12938 | Gh_A05G0184 | Gorai.005G054700 |
|  | AT4G28650 | evm.model.Ga05G1695 | GOBAR_AA12939 | Gh_A05G0352 | Gorai.005G081200 |
|  | AT5G06940 | evm.model.Ga05G1862 | GOBAR_AA12940 | Gh_A05G0399 | Gorai.005G097600 |
|  | AT5G25930 | evm.model.Ga05G1965 | GOBAR_AA13070 | Gh_A05G0446 | Gorai.005G097700 |
|  | AT5G48940 | evm.model.Ga05G2287 | GOBAR_AA13072 | Gh_A05G0447 | Gorai.005G112100 |
|  | AT5G49660 | evm.model.Ga05G2411 | GOBAR_AA13073 | Gh_A05G1341 | Gorai.005G199100 |
|  | AT5G56040 | evm.model.Ga05G2412 | GOBAR_AA13170 | Gh_A05G1591 | Gorai.005G265400 |
|  | AT5G61480 | evm.model.Ga05G2413 | GOBAR_AA13853 | Gh_A05G1951 | Gorai.005G268400 |
|  | AT5G63930 | evm.model.Ga05G2677 | GOBAR_AA13899 | Gh_A05G2137 | Gorai.006G019800 |
|  | AT5G65700 | evm.model.Ga05G2810 | GOBAR_AA14870 | Gh_A05G2259 | Gorai.006G020000 |
|  | AT5G65710 | evm.model.Ga05G2897 | GOBAR_AA17903 | Gh_A05G2324 | Gorai.006G020100 |
|  |  | evm.model.Ga05G2910 | GOBAR_AA18044 | Gh_A05G2332 | Gorai.006G020400 |
|  |  | evm.model.Ga05G4258 | GOBAR_AA19174 | Gh_A05G3781 | Gorai.006G020800 |
|  |  | evm.model.Ga06G0350 | GOBAR_AA19559 | Gh_A06G0228 | Gorai.006G021000 |
|  |  | evm.model.Ga06G0351 | GOBAR_AA19704 | Gh_A06G0229 | Gorai.006G021100 |
|  |  | evm.model.Ga06G0352 | GOBAR_AA21025 | Gh_A06G0291 | Gorai.006G021300 |
|  |  | evm.model.Ga06G1027 | GOBAR_AA21291 | Gh_A06G0292 | Gorai.006G022200 |
|  |  | evm.model.Ga06G1082 | GOBAR_AA21408 | Gh_A06G0294 | Gorai.006G022400 |
|  |  | evm.model.Ga07G0268 | GOBAR_AA21433 | Gh_A06G0824 | Gorai.006G022800 |
|  |  | evm.model.Ga07G0274 | GOBAR_AA21667 | Gh_A06G0865 | Gorai.006G139200 |
|  |  | evm.model.Ga07G0510 | GOBAR_AA22200 | Gh_A07G0162 | Gorai.006G219400 |
|  |  | evm.model.Ga07G1712 | GOBAR_AA22434 | Gh_A07G0363 | Gorai.007G148900 |
|  |  | evm.model.Ga07G1749 | GOBAR_AA22435 | Gh_A07G1429 | Gorai.007G228700 |
|  |  | evm.model.Ga07G2373 | GOBAR_AA23839 | Gh_A07G1863 | Gorai.007G271300 |
|  |  | evm.model.Ga08G1226 | GOBAR_AA24839 | Gh_A07G2223 | Gorai.007G273700 |
|  |  | evm.model.Ga08G2249 | GOBAR_AA25862 | Gh_A07G2299 | Gorai.008G045400 |
|  |  | evm.model.Ga08G2539 | GOBAR_AA27071 | Gh_A08G1699 | Gorai.008G087500 |
|  |  | evm.model.Ga09G0193 | GOBAR_AA29606 | Gh_A08G2329 | Gorai.008G215400 |
|  |  | evm.model.Ga09G0194 | GOBAR_AA29835 | Gh_A09G0186 | Gorai.009G027700 |
|  |  | evm.model.Ga09G0195 | GOBAR_AA30141 | Gh_A09G0187 | Gorai.009G048200 |
|  |  | evm.model.Ga09G0219 | GOBAR_AA31172 | Gh_A09G0196 | Gorai.009G049000 |
|  |  | evm.model.Ga09G0232 | GOBAR_AA31173 | Gh_A09G0197 | Gorai.009G053200 |
|  |  | evm.model.Ga09G0235 | GOBAR_AA31242 | Gh_A09G0199 | Gorai.009G056800 |
|  |  | evm.model.Ga09G0236 | GOBAR_AA32172 | Gh_A09G1169 | Gorai.009G166500 |
|  |  | evm.model.Ga09G0240 | GOBAR_AA32338 | Gh_A09G1800 | Gorai.009G183000 |
|  |  | evm.model.Ga09G0241 | GOBAR_AA33717 | Gh_A10G0070 | Gorai.009G193600 |
|  |  | evm.model.Ga09G1424 | GOBAR_AA34201 | Gh_A10G0261 | Gorai.009G225300 |
|  |  | evm.model.Ga09G2252 | GOBAR_AA34228 | Gh_A10G1074 | Gorai.009G237700 |
|  |  | evm.model.Ga10G0781 | GOBAR_AA34905 | Gh_A10G1681 | Gorai.009G265300 |
|  |  | evm.model.Ga10G0782 | GOBAR_AA36842 | Gh_A10G1689 | Gorai.009G278200 |
|  |  | evm.model.Ga10G0802 | GOBAR_AA37429 | Gh_A11G1221 | Gorai.009G286600 |
|  |  | evm.model.Ga10G0804 | GOBAR_AA37571 | Gh_A11G1901 | Gorai.009G287800 |
|  |  | evm.model.Ga10G0805 | GOBAR_AA37640 | Gh_A11G2202 | Gorai.009G452000 |
|  |  | evm.model.Ga10G0808 | GOBAR_AA39781 | Gh_A11G2215 | Gorai.010G030500 |
|  |  | evm.model.Ga10G0811 | GOBAR_AA39946 | Gh_A12G0410 | Gorai.010G030600 |
|  |  | evm.model.Ga10G1436 | GOBAR_AA40063 | Gh_A12G1795 | Gorai.010G040500 |
|  |  | evm.model.Ga10G2786 | GOBAR_AA40214 | Gh_A12G2553 | Gorai.010G040600 |
|  |  | evm.model.Ga10G3017 | GOBAR_AA40857 | Gh_D01G0764 | Gorai.010G040900 |
|  |  | evm.model.Ga11G1130 | GOBAR_DD00003 | Gh_D01G1753 | Gorai.010G041500 |
|  |  | evm.model.Ga11G1201 | GOBAR_DD00005 | Gh_D01G1801 | Gorai.010G041800 |
|  |  | evm.model.Ga11G1756 | GOBAR_DD01734 | Gh_D02G0338 | Gorai.010G104300 |
|  |  | evm.model.Ga11G2645 | GOBAR_DD02092 | Gh_D02G0479 | Gorai.010G110800 |
|  |  | evm.model.Ga12G0830 | GOBAR_DD02282 | Gh_D02G0724 | Gorai.011G008200 |
|  |  | evm.model.Ga12G2129 | GOBAR_DD02711 | Gh_D02G0867 | Gorai.011G030400 |
|  |  | evm.model.Ga12G2660 | GOBAR_DD04499 | Gh_D02G0868 | Gorai.011G162100 |
|  |  | evm.model.Ga14G1493 | GOBAR_DD04512 | Gh_D02G0981 | Gorai.011G217500 |
|  |  | evm.model.Ga14G1495 | GOBAR_DD05650 | Gh_D02G1808 | Gorai.011G217600 |
|  |  | evm.model.Ga14G1601 | GOBAR_DD06185 | Gh_D02G2351 | Gorai.011G217700 |
|  |  |  | GOBAR_DD06690 | Gh_D02G2378 | Gorai.011G217800 |
|  |  |  | GOBAR_DD06691 | Gh_D03G0300 | Gorai.011G218200 |
|  |  |  | GOBAR_DD06693 | Gh_D03G0302 | Gorai.011G218900 |
|  |  |  | GOBAR_DD06848 | Gh_D03G0303 | Gorai.011G219000 |
|  |  |  | GOBAR_DD06849 | Gh_D05G0258 | Gorai.011G219300 |
|  |  |  | GOBAR_DD06852 | Gh_D05G0470 | Gorai.N025300 |
|  |  |  | GOBAR_DD06854 | Gh_D05G0478 |  |
|  |  |  | GOBAR_DD06855 | Gh_D05G0548 |  |
|  |  |  | GOBAR_DD06857 | Gh_D05G0551 |  |
|  |  |  | GOBAR_DD07118 | Gh_D05G1510 |  |
|  |  |  | GOBAR_DD09695 | Gh_D05G1768 |  |
|  |  |  | GOBAR_DD10329 | Gh_D05G2072 |  |
|  |  |  | GOBAR_DD11685 | Gh_D05G2179 |  |
|  |  |  | GOBAR_DD13422 | Gh_D05G2391 |  |
|  |  |  | GOBAR_DD13423 | Gh_D05G2518 |  |
|  |  |  | GOBAR_DD14531 | Gh_D05G2519 |  |
|  |  |  | GOBAR_DD15105 | Gh_D05G2585 |  |
|  |  |  | GOBAR_DD15432 | Gh_D05G2594 |  |
|  |  |  | GOBAR_DD15434 | Gh_D05G3696 |  |
|  |  |  | GOBAR_DD15435 | Gh_D06G0311 |  |
|  |  |  | GOBAR_DD15472 | Gh_D06G0952 |  |
|  |  |  | GOBAR_DD15516 | Gh_D06G1009 |  |
|  |  |  | GOBAR_DD16325 | Gh_D06G2311 |  |
|  |  |  | GOBAR_DD16333 | Gh_D07G0212 |  |
|  |  |  | GOBAR_DD16433 | Gh_D07G0219 |  |
|  |  |  | GOBAR_DD17201 | Gh_D07G0425 |  |
|  |  |  | GOBAR_DD17870 | Gh_D07G1527 |  |
|  |  |  | GOBAR_DD18730 | Gh_D07G1551 |  |
|  |  |  | GOBAR_DD19037 | Gh_D07G2075 |  |
|  |  |  | GOBAR_DD20631 | Gh_D08G1121 |  |
|  |  |  | GOBAR_DD20844 | Gh_D08G2057 |  |
|  |  |  | GOBAR_DD20854 | Gh_D08G2304 |  |
|  |  |  | GOBAR_DD21847 | Gh_D09G0166 |  |
|  |  |  | GOBAR_DD21848 | Gh_D09G0167 |  |
|  |  |  | GOBAR_DD22853 | Gh_D09G0172 |  |
|  |  |  | GOBAR_DD22993 | Gh_D09G0173 |  |
|  |  |  | GOBAR_DD23834 | Gh_D09G0174 |  |
|  |  |  | GOBAR_DD24011 | Gh_D09G0176 |  |
|  |  |  | GOBAR_DD24609 | Gh_D09G0177 |  |
|  |  |  | GOBAR_DD25778 | Gh_D09G0186 |  |
|  |  |  | GOBAR_DD25935 | Gh_D09G1175 |  |
|  |  |  | GOBAR_DD25962 | Gh_D09G1921 |  |
|  |  |  | GOBAR_DD26344 | Gh_D10G0074 |  |
|  |  |  | GOBAR_DD26345 | Gh_D10G0261 |  |
|  |  |  | GOBAR_DD27423 | Gh_D10G1439 |  |
|  |  |  | GOBAR_DD27426 | Gh_D10G1934 |  |
|  |  |  | GOBAR_DD27821 | Gh_D10G1940 |  |
|  |  |  | GOBAR_DD27855 | Gh_D10G1941 |  |
|  |  |  | GOBAR_DD30021 | Gh_D10G1956 |  |
|  |  |  | GOBAR_DD30919 | Gh_D11G1367 |  |
|  |  |  | GOBAR_DD31049 | Gh_D11G2071 |  |
|  |  |  | GOBAR_DD32016 | Gh_D11G2499 |  |
|  |  |  | GOBAR_DD32097 | Gh_D11G2524 |  |
|  |  |  | GOBAR_DD32099 | Gh_D12G0403 |  |
|  |  |  | GOBAR_DD35462 | Gh_D12G0758 |  |
|  |  |  | GOBAR_DD35710 | Gh_D12G1963 |  |
|  |  |  | GOBAR_DD36049 | Gh_Sca007876G01 |  |
|  |  |  | GOBAR_DD38131 |  |  |
|  |  |  | GOBAR_DD38159 |  |  |
|  |  |  | GOBAR_DD38304 |  |  |
| XI-2 | AT1G34420 | evm.model.Ga02G0751 | GOBAR_DD02552 | Gh_A02G1065 | Gorai.003G092400 |
|  | AT2G41820 | evm.model.Ga09G1343 | GOBAR_DD26121 | Gh_A09G1099 | Gorai.006G131800 |
|  |  | evm.model.Ga09G1403 | GOBAR_DD29900 | Gh_A09G1150 | Gorai.006G137100 |
|  |  |  |  | Gh_D03G0613 |  |
|  |  |  |  | Gh_D09G1105 |  |
|  |  |  |  | Gh_D09G1153 |  |
|  |  |  |  | Gh_D09G1156 |  |
| XI-3 | AT4G20140 | evm.model.Ga12G2605 | GOBAR_DD10462 | Gh_A12G0463 | Gorai.008G051700 |
|  | AT5G44700 |  |  | Gh_D12G0466 |  |
| XII | AT2G24130 | evm.model.Ga01G0489 | GOBAR_AA01931 | Gh_A01G0330 | Gorai.002G049100 |
|  | AT3G47090 | evm.model.Ga01G0495 | GOBAR_AA01932 | Gh_A01G0355 | Gorai.002G049400 |
|  | AT3G47110 | evm.model.Ga01G0522 | GOBAR_AA01934 | Gh_A01G0412 | Gorai.002G051100 |
|  | AT3G47570 | evm.model.Ga01G0523 | GOBAR_AA01938 | Gh_A01G0988 | Gorai.002G051500 |
|  | AT3G47580 | evm.model.Ga01G0601 | GOBAR_AA01940 | Gh_A01G1935 | Gorai.002G051700 |
|  | AT5G20480 | evm.model.Ga01G1423 | GOBAR_AA01942 | Gh_A04G0722 | Gorai.002G051900 |
|  | AT5G46330 | evm.model.Ga04G0709 | GOBAR_AA01946 | Gh_A04G1361 | Gorai.002G064300 |
|  |  | evm.model.Ga04G1059 | GOBAR_AA03351 | Gh_A05G0746 | Gorai.002G131000 |
|  |  | evm.model.Ga05G0944 | GOBAR_AA03354 | Gh_A05G2502 | Gorai.002G133000 |
|  |  | evm.model.Ga05G3122 | GOBAR_AA04744 | Gh_A05G2555 | Gorai.002G260200 |
|  |  | evm.model.Ga05G3124 | GOBAR_AA04801 | Gh_A05G2753 | Gorai.004G027800 |
|  |  | evm.model.Ga05G3134 | GOBAR_AA06734 | Gh_A06G1026 | Gorai.007G045900 |
|  |  | evm.model.Ga05G3203 | GOBAR_AA12358 | Gh_A06G1191 | Gorai.007G181000 |
|  |  | evm.model.Ga05G3204 | GOBAR_AA14525 | Gh_A08G2505 | Gorai.008G290200 |
|  |  | evm.model.Ga05G3551 | GOBAR_AA15904 | Gh_A10G0003 | Gorai.009G092700 |
|  |  | evm.model.Ga05G3802 | GOBAR_AA18116 | Gh_A10G0350 | Gorai.009G237800 |
|  |  | evm.model.Ga05G3803 | GOBAR_AA18634 | Gh_A10G1844 | Gorai.009G306900 |
|  |  | evm.model.Ga05G3805 | GOBAR_AA19796 | Gh_A10G1953 | Gorai.009G307100 |
|  |  | evm.model.Ga05G3809 | GOBAR_AA20233 | Gh_A10G1956 | Gorai.009G308400 |
|  |  | evm.model.Ga05G3832 | GOBAR_AA20954 | Gh_A10G2098 | Gorai.009G313800 |
|  |  | evm.model.Ga05G4128 | GOBAR_AA21772 | Gh_A10G2099 | Gorai.009G314000 |
|  |  | evm.model.Ga06G1344 | GOBAR_AA22072 | Gh_A10G2101 | Gorai.009G340400 |
|  |  | evm.model.Ga06G1653 | GOBAR_AA22384 | Gh_A10G2102 | Gorai.009G365300 |
|  |  | evm.model.Ga08G0283 | GOBAR_AA22404 | Gh_A10G2103 | Gorai.009G366000 |
|  |  | evm.model.Ga10G0014 | GOBAR_AA23965 | Gh_A10G2104 | Gorai.009G366200 |
|  |  | evm.model.Ga10G0015 | GOBAR_AA24583 | Gh_A11G1506 | Gorai.009G369800 |
|  |  | evm.model.Ga10G0020 | GOBAR_AA29975 | Gh_D01G0371 | Gorai.009G370000 |
|  |  | evm.model.Ga10G0022 | GOBAR_AA31096 | Gh_D01G0386 | Gorai.009G370300 |
|  |  | evm.model.Ga10G0024 | GOBAR_AA31100 | Gh_D01G0417 | Gorai.009G371100 |
|  |  | evm.model.Ga10G0026 | GOBAR_AA31997 | Gh_D01G1037 | Gorai.009G371300 |
|  |  | evm.model.Ga10G0028 | GOBAR_AA32005 | Gh_D04G0846 | Gorai.009G371600 |
|  |  | evm.model.Ga10G0029 | GOBAR_AA32006 | Gh_D05G2770 | Gorai.009G435400 |
|  |  | evm.model.Ga10G0031 | GOBAR_AA32008 | Gh_D05G2831 | Gorai.009G436200 |
|  |  | evm.model.Ga10G0318 | GOBAR_AA32009 | Gh_D05G2832 | Gorai.009G436300 |
|  |  | evm.model.Ga10G0323 | GOBAR_AA32525 | Gh_D05G3056 | Gorai.010G136200 |
|  |  | evm.model.Ga10G0324 | GOBAR_AA34940 | Gh_D05G3282 | Gorai.010G165300 |
|  |  | evm.model.Ga10G0325 | GOBAR_AA35311 | Gh_D05G3320 | Gorai.011G000900 |
|  |  | evm.model.Ga10G0326 | GOBAR_AA35555 | Gh_D05G3552 | Gorai.011G181600 |
|  |  | evm.model.Ga10G0327 | GOBAR_AA36259 | Gh_D05G3554 | Gorai.011G239600 |
|  |  | evm.model.Ga10G0338 | GOBAR_AA36263 | Gh_D05G3556 | Gorai.011G258200 |
|  |  | evm.model.Ga10G0340 | GOBAR_AA36264 | Gh_D05G3751 | Gorai.011G258400 |
|  |  | evm.model.Ga10G0341 | GOBAR_AA40301 | Gh_D05G3754 | Gorai.011G258500 |
|  |  | evm.model.Ga10G0546 | GOBAR_AA40652 | Gh_D06G2353 | Gorai.011G258700 |
|  |  | evm.model.Ga10G2685 | GOBAR_AA40708 | Gh_D08G0243 | Gorai.011G259100 |
|  |  | evm.model.Ga10G3089 | GOBAR_AA40710 | Gh_D10G0004 | Gorai.011G259200 |
|  |  | evm.model.Ga11G2293 | GOBAR_AA40711 | Gh_D10G0357 | Gorai.011G259300 |
|  |  | evm.model.Ga11G3667 | GOBAR_AA40712 | Gh_D10G2230 | Gorai.011G259500 |
|  |  | evm.model.Ga12G0082 | GOBAR_DD01141 | Gh_D10G2231 | Gorai.011G259800 |
|  |  | evm.model.Ga14G0100 | GOBAR_DD01159 | Gh_D10G2234 | Gorai.011G260200 |
|  |  |  | GOBAR_DD01401 | Gh_D10G2235 | Gorai.011G260300 |
|  |  |  | GOBAR_DD04536 | Gh_D10G2236 | Gorai.011G284100 |
|  |  |  | GOBAR_DD06238 | Gh_D10G2239 | Gorai.011G284200 |
|  |  |  | GOBAR_DD06552 | Gh_D10G2240 | Gorai.011G284300 |
|  |  |  | GOBAR_DD10689 | Gh_D10G2368 | Gorai.011G284400 |
|  |  |  | GOBAR_DD13217 | Gh_D10G2373 | Gorai.011G284600 |
|  |  |  | GOBAR_DD13219 | Gh_D10G2376 | Gorai.011G284900 |
|  |  |  | GOBAR_DD13221 | Gh_D10G2379 | Gorai.011G285100 |
|  |  |  | GOBAR_DD13223 | Gh_D10G2380 | Gorai.011G285300 |
|  |  |  | GOBAR_DD13225 | Gh_D11G0425 | Gorai.011G285400 |
|  |  |  | GOBAR_DD13226 | Gh_D11G1668 | Gorai.011G285500 |
|  |  |  | GOBAR_DD13785 | Gh_D12G2558 | Gorai.012G098700 |
|  |  |  | GOBAR_DD13788 |  | Gorai.012G106100 |
|  |  |  | GOBAR_DD13790 |  | Gorai.N016200 |
|  |  |  | GOBAR_DD13791 |  |  |
|  |  |  | GOBAR_DD13798 |  |  |
|  |  |  | GOBAR_DD14277 |  |  |
|  |  |  | GOBAR_DD14286 |  |  |
|  |  |  | GOBAR_DD16061 |  |  |
|  |  |  | GOBAR_DD18273 |  |  |
|  |  |  | GOBAR_DD18286 |  |  |
|  |  |  | GOBAR_DD20172 |  |  |
|  |  |  | GOBAR_DD20399 |  |  |
|  |  |  | GOBAR_DD21228 |  |  |
|  |  |  | GOBAR_DD21606 |  |  |
|  |  |  | GOBAR_DD21612 |  |  |
|  |  |  | GOBAR_DD22895 |  |  |
|  |  |  | GOBAR_DD23061 |  |  |
|  |  |  | GOBAR_DD23064 |  |  |
|  |  |  | GOBAR_DD23066 |  |  |
|  |  |  | GOBAR_DD23068 |  |  |
|  |  |  | GOBAR_DD23406 |  |  |
|  |  |  | GOBAR_DD24035 |  |  |
|  |  |  | GOBAR_DD24037 |  |  |
|  |  |  | GOBAR_DD24038 |  |  |
|  |  |  | GOBAR_DD24040 |  |  |
|  |  |  | GOBAR_DD24044 |  |  |
|  |  |  | GOBAR_DD24595 |  |  |
|  |  |  | GOBAR_DD24596 |  |  |
|  |  |  | GOBAR_DD25564 |  |  |
|  |  |  | GOBAR_DD29224 |  |  |
|  |  |  | GOBAR_DD29328 |  |  |
|  |  |  | GOBAR_DD30211 |  |  |
|  |  |  | GOBAR_DD30452 |  |  |
|  |  |  | GOBAR_DD32616 |  |  |
|  |  |  | GOBAR_DD32621 |  |  |
|  |  |  | GOBAR_DD32622 |  |  |
|  |  |  | GOBAR_DD33183 |  |  |
|  |  |  | GOBAR_DD33185 |  |  |
|  |  |  | GOBAR_DD34040 |  |  |
|  |  |  | GOBAR_DD35196 |  |  |
|  |  |  | GOBAR_DD36796 |  |  |
|  |  |  | GOBAR_DD37202 |  |  |
| XIII-1 | AT1G31420 | evm.model.Ga07G0764 | GOBAR_AA29177 | Gh_A07G0591 | Gorai.001G074000 |
|  | AT2G35620 | evm.model.Ga09G2236 | GOBAR_AA29370 | Gh_A09G1783 | Gorai.007G035200 |
|  | AT5G62710 | evm.model.Ga11G3780 | GOBAR_AA34607 | Gh_A11G3010 |  |
|  |  |  | GOBAR_DD09192 | Gh_D09G1906 |  |
|  |  |  | GOBAR_DD21585 | Gh_D11G0315 |  |
|  |  |  | GOBAR_DD23720 |  |  |
| XIII-2 | AT2G26330 | evm.model.Ga06G0937 | GOBAR_AA11772 | Gh_A06G0747 | Gorai.001G019800 |
|  | AT5G07180 | evm.model.Ga07G0208 | GOBAR_AA18091 | Gh_A07G2348 | Gorai.007G009000 |
|  | AT5G62230 | evm.model.Ga11G4047 | GOBAR_AA36304 | Gh_A11G0071 | Gorai.010G096300 |
|  |  |  | GOBAR_DD04068 | Gh_D06G0879 |  |
|  |  |  | GOBAR_DD20603 | Gh_D07G0162 |  |
|  |  |  | GOBAR_DD25858 | Gh_D11G0073 |  |
| XIV | AT2G16250 | evm.model.Ga03G2525 | GOBAR_AA02368 | Gh_A01G0939 | Gorai.002G127700 |
|  | AT4G39270 | evm.model.Ga06G0111 | GOBAR_AA02674 | Gh_A03G2177 | Gorai.005G243800 |
|  |  | evm.model.Ga13G0603 | GOBAR_AA17358 | Gh_A06G0079 | Gorai.008G029000 |
|  |  | evm.model.Ga14G0201 | GOBAR_AA25693 | Gh_A12G0256 | Gorai.010G010100 |
|  |  | evm.model.Ga14G0349 | GOBAR_AA29303 | Gh_A13G0295 | Gorai.013G036900 |
|  |  |  | GOBAR_DD00263 | Gh_D01G0986 |  |
|  |  |  | GOBAR_DD03868 | Gh_D02G2145 |  |
|  |  |  | GOBAR_DD19141 | Gh_D12G0257 |  |
|  |  |  | GOBAR_DD24411 | Gh_D13G0333 |  |
|  |  |  | GOBAR_DD35623 |  |  |
| XV | AT1G69270 | evm.model.Ga01G2008 | GOBAR_AA12037 | Gh_A03G0563 | Gorai.001G149100 |
|  | AT2G31880 | evm.model.Ga09G0143 | GOBAR_AA15875 | Gh_A07G1209 | Gorai.003G108000 |
|  | AT3G02130 | evm.model.Ga11G3303 | GOBAR_AA20166 | Gh_A09G0087 | Gorai.006G010100 |
|  |  | evm.model.Ga11G3404 | GOBAR_AA27335 | Gh_A11G0593 | Gorai.007G073100 |
|  |  | evm.model.Ga12G0961 | GOBAR_AA36270 | Gh_A11G0659 | Gorai.007G083000 |
|  |  | evm.model.Ga12G1232 | GOBAR_DD01583 | Gh_A12G1475 | Gorai.008G176800 |
|  |  | evm.model.Ga13G0995 | GOBAR_DD05211 | Gh_A12G1689 | Gorai.008G203300 |
|  |  | evm.model.Ga14G2013 | GOBAR_DD06254 | Gh_D03G0968 | Gorai.013G094500 |
|  |  |  | GOBAR_DD06732 | Gh_D07G1311 |  |
|  |  |  | GOBAR_DD11376 | Gh_D09G0084 |  |
|  |  |  | GOBAR_DD21385 | Gh_D11G0680 |  |
|  |  |  | GOBAR_DD28204 | Gh_D11G0772 |  |
|  |  |  | GOBAR_DD28441 | Gh_D12G1600 |  |
|  |  |  |  | Gh_D12G1850 |  |
|  |  |  |  | Gh_D13G0859 |  |

**Table S2**

| **Chromosome** | **Number (percentage) of *LRR-RLK* genes** | | | |
| --- | --- | --- | --- | --- |
|  | ***G. arboreum*** | ***G. barbadense*** | ***G. hirsutum*** | ***G. raimondii*** |
| A01 | 18(6.0%) | 14(2.7%) | 18(3.5%) | - |
| A02 | 14(4.7%) | 15(2.9%) | 19(3.7%) | - |
| A03 | 18(6.0%) | 11(2.2%) | 11(2.1%) | - |
| A04 | 6(2.0%) | 11(2.2%) | 6(1.2%) | - |
| A05 | 48(16.1%) | 31(6.1%) | 28(5.4%) | - |
| A06 | 24(8.1%) | 23(4.5%) | 20(3.9%) | - |
| A07 | 19(6.4%) | 13(2.5%) | 14(2.7%) | - |
| A08 | 11(3.7%) | 6(1.2%) | 7(1.4%) | - |
| A09 | 25(8.4%) | 19(3.7%) | 19(3.7%) | - |
| A10 | 49(16.4%) | 36(7.0%) | 31(6.0%) | - |
| A11 | 26(8.7%) | 21(4.1%) | 21(4.1%) | - |
| A12 | 16(5.4%) | 11(2.2%) | 15(2.9%) | - |
| A13 | 13(4.4%) | 11(2.2%) | 9(1.7%) | - |
| D01 | - | 24(4.7%) | 19(3.7%) | 23(7.3%) |
| D02 | - | 18(3.5%) | 17(3.3%) | 26(8.2%) |
| D03 | - | 11(2.2%) | 11(2.1%) | 12(3.8%) |
| D04 | - | 5(1.0%) | 6(1.2%) | 10(3.2%) |
| D05 | - | 51(10.0%) | 40(7.8%) | 20(6.3%) |
| D06 | - | 27(5.3%) | 19(3.7%) | 24(7.6%) |
| D07 | - | 18(3.5%) | 18(3.5%) | 26(8.2%) |
| D08 | - | 9(1.8%) | 11(2.1%) | 18(5.7%) |
| D09 | - | 24(4.7%) | 25(4.9%) | 52(16.4%) |
| D10 | - | 34(6.7%) | 38(7.4%) | 30(9.5%) |
| D11 | - | 25(4.9%) | 24(4.7%) | 52(16.4%) |
| D12 | - | 12(2.3%) | 16(3.1%) | 7(2.2%) |
| D13 | - | 12(2.3%) | 13(2.5%) | 13(4.1%) |
| scaffolds | 11(3.7%) | 19(3.7%) | 40(7.8%) | 4(1.3%) |
| **Total** | **298(100%)** | **511(100%)** | **515(100%)** | **317(100%)** |

**Table S3**

| **Subfamily** | **Number of tandem duplication genes** | | | |
| --- | --- | --- | --- | --- |
|  | ***G. arboreum*** | ***G. barbadense*** | ***G. hirsutum*** | ***G. raimondii*** |
| II | 5 (29.4%) | 2 (8.7%) | 5 (17.2%) | 5 (27.8%) |
| III | 2 (4.4%) | 4 (5.9%) | 4 (4.8%) | 2 (4.3%) |
| IX |  | 2 (11.1%) |  |  |
| VII-1 |  | 4 (36.4%) |  |  |
| VIII-2 | 10 (58.8%) | 11 (42.3%) | 14 (46.7%) | 11 (64.7%) |
| XI-1 | 31 (41.3%) | 59 (43.7%) | 42 (31.8%) | 35 (43.2%) |
| XI-2 |  |  | 2 (28.6%) |  |
| XII | 31 (63.3%) | 64 (62.7%) | 25 (41.0%) | 43 (68.3%) |

**Table S4**

| ***cis*-acting regulatory element name  (ID of CARE)** | **Number of elements found in promoters of *LRR-RLK* genes** | | | |
| --- | --- | --- | --- | --- |
|  | ***G. arboreum*** | ***G. barbadense*** | ***G. hirsutum*** | ***G. raimondii*** |
| CAAT-box | 298 | 509 | 515 | 310 |
| TATA-box | 298 | 504 | 514 | 309 |
| AT-TATA-box | 243 | 413 | 407 | 265 |
| TATA | 169 | 268 | 256 | 178 |
| A-box | 25 | 42 | 48 | 23 |
| Myb | 285 | 469 | 492 | 288 |
| AT-rich element | 41 | 71 | 62 | 55 |
| MYC | 279 | 450 | 479 | 274 |
| as-1 | 149 | 216 | 228 | 150 |
| MBS | 101 | 166 | 196 | 95 |
| DRE core | 17 | 28 | 33 | 24 |
| ACTCATCCT sequence | 5 | 6 | 6 | 3 |
| DRE1 | 12 | 18 | 17 | 10 |
| MYB recognition site | 50 | 69 | 76 | 57 |
| LTR | 79 | 150 | 168 | 98 |
| STRE | 202 | 337 | 347 | 177 |
| TCA | 80 | 133 | 143 | 68 |
| ARE | 236 | 378 | 402 | 229 |
| GC-motif | 16 | 34 | 32 | 23 |
| TC-rich repeats | 110 | 167 | 181 | 108 |
| W box | 149 | 256 | 252 | 158 |
| WUN-motif | 128 | 209 | 213 | 128 |
| WRE3 | 90 | 157 | 173 | 94 |
| box S | 27 | 42 | 45 | 23 |
| W box | 149 | 256 | 252 | 158 |
| GT1-motif | 178 | 296 | 305 | 192 |
| TCT-motif | 144 | 237 | 248 | 130 |
| GATA-motif | 117 | 184 | 191 | 113 |
| MRE | 92 | 136 | 141 | 95 |
| AE-box | 86 | 144 | 167 | 76 |
| I-box | 69 | 111 | 103 | 65 |
| TCCC-motif | 62 | 88 | 106 | 59 |
| ATCT-motif | 59 | 93 | 95 | 55 |
| AT1-motif | 49 | 75 | 77 | 44 |
| GA-motif | 47 | 84 | 74 | 53 |
| chs-CMA1a | 46 | 98 | 99 | 57 |
| LAMP-element | 32 | 56 | 59 | 27 |
| 3-AF1 binding site | 29 | 44 | 48 | 28 |
| ACE | 24 | 50 | 48 | 45 |
| Gap-box | 23 | 24 | 31 | 15 |
| Sp1 | 23 | 42 | 52 | 32 |
| chs-CMA2a | 19 | 32 | 27 | 16 |
| ATC-motif | 13 | 21 | 28 | 14 |
| Box II | 12 | 26 | 28 | 21 |
| AAAC-motif | 11 | 10 | 12 | 6 |
| GTGGC-motif | 7 | 8 | 10 | 3 |
| CAG-motif | 5 | 9 | 8 | 9 |
| ACA-motif | 4 | 8 | 9 | 9 |
| chs-Unit 1 m1 | 3 | 5 | 5 | 5 |
| L-box | 3 | 9 | 8 | 5 |
| LS7 | 2 | 6 | 4 | 4 |
| 4cl-CMA2b | 1 | 2 | 2 | 1 |
| Pc-CMA2c | 1 | 2 | 2 | 2 |
| circadian | 36 | 83 | 79 | 52 |
| ERE | 226 | 354 | 370 | 242 |
| ABRE | 160 | 268 | 299 | 175 |
| ABRE3a | 56 | 81 | 101 | 68 |
| ABRE4 | 56 | 81 | 101 | 68 |
| ABRE2 | 2 | 3 | 6 | 2 |
| AT-ABRE | 1 | 6 | 5 | 6 |
| P-box | 68 | 117 | 130 | 68 |
| TATC-box | 43 | 63 | 70 | 41 |
| GARE-motif | 39 | 59 | 65 | 35 |
| CARE | 19 | 39 | 40 | 21 |
| CGTCA-motif | 149 | 216 | 228 | 150 |
| TGACG-motif | 149 | 216 | 228 | 150 |
| JERE | 2 | 3 | 4 | 2 |
| TCA-element | 133 | 191 | 218 | 112 |
| TGA-element | 60 | 93 | 87 | 71 |
| TGA-box | 10 | 11 | 12 | 10 |
| AuxRR-core | 23 | 50 | 48 | 35 |
| AuxRE | 1 | 1 | 2 | 2 |
| Myb-binding site | 116 | 197 | 200 | 112 |
| CCGTCC motif | 24 | 40 | 45 | 21 |
| MSA-like | 11 | 20 | 18 | 9 |
| re2f-1 | 4 | 7 | 4 | 3 |
| NON | 3 | 3 | 5 | 3 |
| dOCT | 3 | 6 | 7 | 3 |
| E2Fb | 2 | 4 | 7 | 4 |
| O2-site | 80 | 126 | 137 | 80 |
| MBSI | 28 | 41 | 39 | 24 |
| AP-1 | 17 | 22 | 27 | 16 |
| CAT-box | 71 | 128 | 132 | 89 |
| MYB-like sequence | 171 | 283 | 299 | 166 |
| GCN4_motif | 49 | 66 | 73 | 42 |
| RY-element | 13 | 20 | 26 | 17 |
| AC-I | 10 | 9 | 14 | 6 |
| AC-II | 5 | 8 | 9 | 3 |
| AACA_motif | 3 | 3 | 7 | 3 |
| telo-box | 2 | 2 | 5 | 2 |
| motif I | 1 | 1 | 2 | 3 |
| Box III | 20 | 19 | 26 | 14 |
| HD-Zip 3 | 20 | 21 | 23 | 10 |
| AT-rich sequence | 25 | 33 | 30 | 25 |
| HD-Zip 1 | 15 | 24 | 24 | 13 |
| Unnamed__4 | 293 | 499 | 510 | 304 |
| Unnamed__6 | 69 | 105 | 117 | 74 |
| Unnamed__1 | 136 | 252 | 247 | 146 |
| Unnamed__2 | 34 | 55 | 57 | 38 |
| Unnamed__10 | 6 | 14 | 14 | 15 |
| Unnamed__12 | 6 | 14 | 14 | 15 |
| Unnamed__14 | 6 | 14 | 14 | 15 |
| Unnamed__8 | 6 | 14 | 14 | 15 |
| Unnamed__16 | 2 | 1 | 1 | 1 |
| Unnamed__3 | 1 | 1 | 3 | 3 |
| AAGAA-motif | 210 | 339 | 373 | 186 |
| CCAAT-box | 50 | 69 | 76 | 57 |
| CCGTCC-box | 24 | 40 | 45 | 21 |
| F-box | 22 | 32 | 40 | 21 |
| CTAG-motif | 21 | 45 | 42 | 23 |
| 3-AF3 binding site | 9 | 7 | 13 | 6 |
| Box II -like sequence | 1 | 2 | 3 | 1 |

**Table S5**

| **TF family** | **Number of TFs regulated *LRR-RLK* gene** | | |
| --- | --- | --- | --- |
|  | ***G. arboreum*** | ***G. hirsutum*** | ***G. raimondii*** |
| AP2 | 188 | 335 | 159 |
| ARF | 35 | 83 | 49 |
| ARR-B | 8 | 10 | 3 |
| B3 | 42 | 78 | 58 |
| BBR-BPC | 137 | 275 | 107 |
| BES1 | 23 | 42 | 35 |
| bHLH | 98 | 197 | 106 |
| bZIP | 73 | 144 | 82 |
| C2H2 | 172 | 315 | 172 |
| C3H | 6 | 13 | 6 |
| CAMTA | 15 | 28 | 20 |
| CPP | 19 | 43 | 36 |
| Dof | 231 | 418 | 215 |
| E2F/DP | 11 | 18 | 10 |
| EIL | 13 | 29 | 14 |
| ERF | 156 | 301 | 150 |
| FAR1 | 21 | 20 | 14 |
| G2-like | 57 | 114 | 67 |
| GATA | 99 | 168 | 84 |
| GRAS | 132 | 244 | 97 |
| HD-ZIP | 62 | 142 | 62 |
| HSF | 25 | 38 | 20 |
| LBD | 63 | 123 | 59 |
| LFY | 3 | 4 | 4 |
| MIKC_MADS | 213 | 373 | 193 |
| MYB | 195 | 354 | 200 |
| MYB_related | 49 | 93 | 63 |
| NAC | 100 | 169 | 87 |
| Nin-like | 44 | 115 | 41 |
| RAV | 14 | 25 | 14 |
| SBP | 13 | 33 | 19 |
| SRS | 4 | 9 | 4 |
| TALE | 107 | 195 | 89 |
| TCP | 101 | 164 | 114 |
| Trihelix | 84 | 141 | 77 |
| WOX | 18 | 41 | 30 |
| WRKY | 93 | 150 | 83 |
| YABBY | 2 | 6 | 5 |
| ZF-HD | 28 | 21 | 22 |
