# Supplementary material for: Genome-Wide Analysis of LRR-RLK Gene Family in Four Gossypium Species and Expression Analysis during Cotton Development and Stress Responses
Source: Genes (Basel). 2018 Nov 29;9(12):592. doi: 10.3390/genes9120592 (PMC6316826; doi:10.3390/genes9120592)

Figure S2: NJ tree constructed by MEGA 7 based on LRR-RLK family members of *A. thaliana* and *Gossypium*.

Amino acid sequences of LRR-RLKs from *A. thaliana* and *Gossypium* were aligned by MUSCLE software with default parameters. Aligned sequenced were used to constructed NJ tree by MEGA 7 with 1000 bootstraps. Only topology of tree was display. Bootstrap alues lower than 50 were hidden.

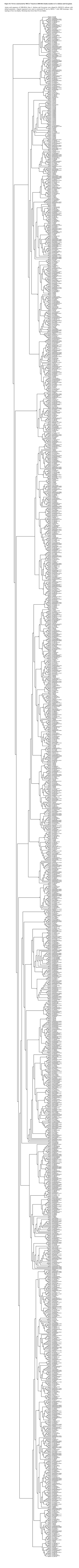

Supplement: Supplementary file 1 [file genes-09-00592-s001.zip › Supplementary Materials (Figure S2).pdf]
