# Supplementary material for: Genome-Wide Analysis of LRR-RLK Gene Family in Four Gossypium Species and Expression Analysis during Cotton Development and Stress Responses
Source: Genes (Basel). 2018 Nov 29;9(12):592. doi: 10.3390/genes9120592 (PMC6316826; doi:10.3390/genes9120592)

Figure S3: Chromosomal location of *LRR-RLK* genes from four *Gossypium* species.

LRR-RLK genes from *G. arboreum*, *G. barbadense*, *G. hirsutum*, *G. raimondii* were mapped to chromosomes based on chromosomal coordinates extracted from gene feature annotation files (shown by A, B, C, D, respectively). *LRR-RLK* genes located on scaffolds were not shown. Tandem duplication gene sets were highlighted by red border rectangles to represent.

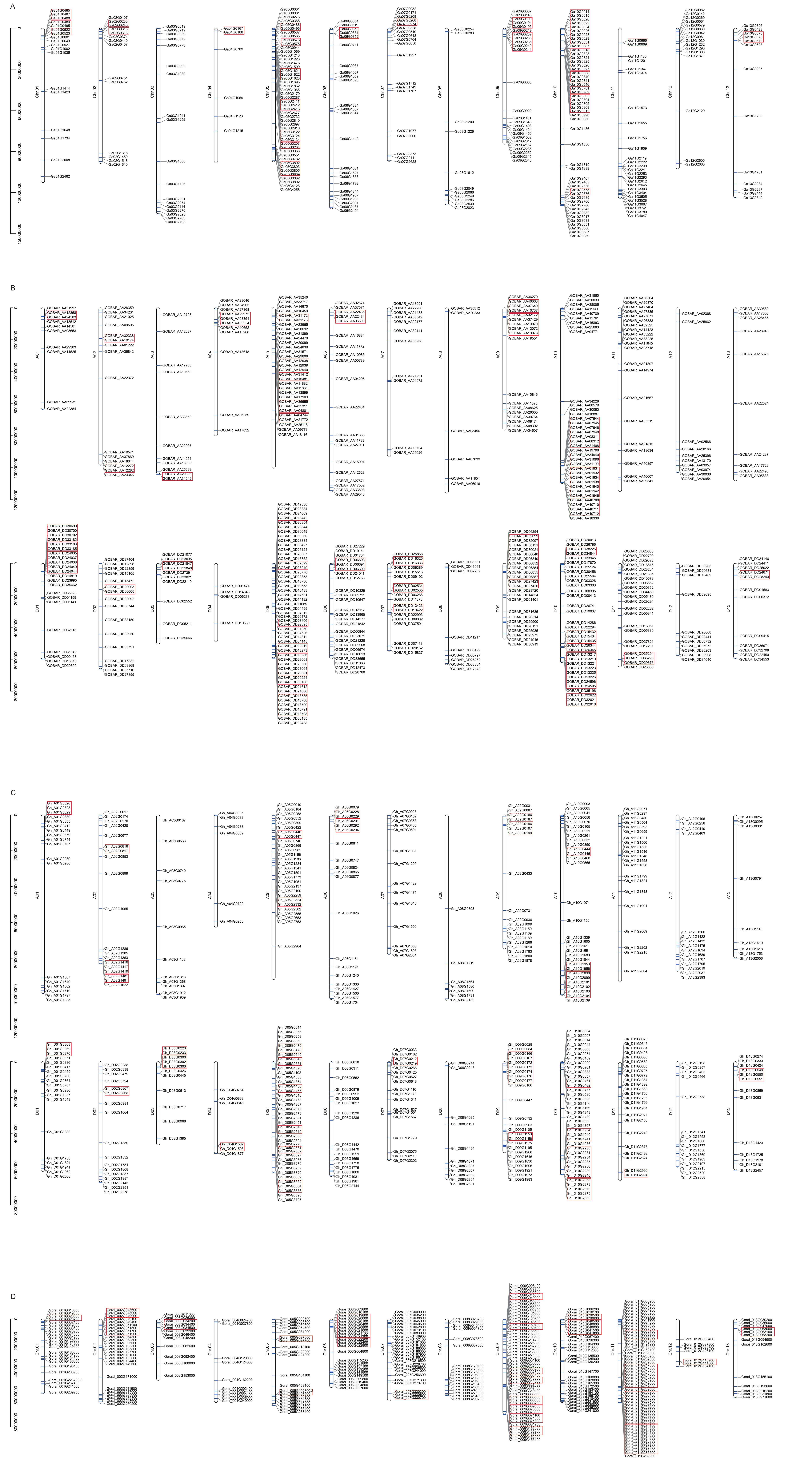

Supplement: Supplementary file 1 [file genes-09-00592-s001.zip › Supplementary Materials (Figure S3).pdf]
