# Supplementary figures and images for: Genome-Wide Analysis of LRR-RLK Gene Family in Four Gossypium Species and Expression Analysis during Cotton Development and Stress Responses
Source: Genes (Basel). 2018 Nov 29;9(12):592. doi: 10.3390/genes9120592 (PMC6316826; doi:10.3390/genes9120592)

A

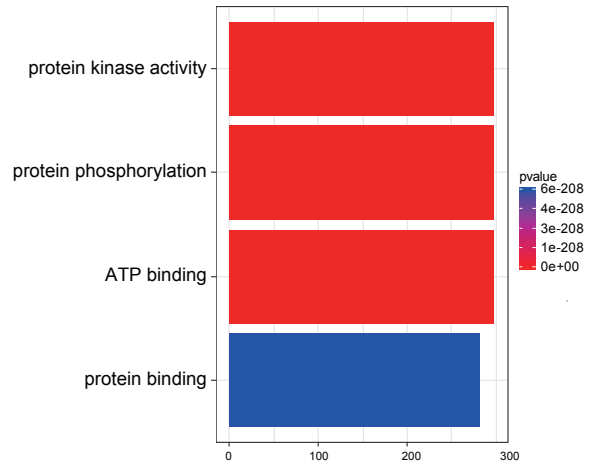

B

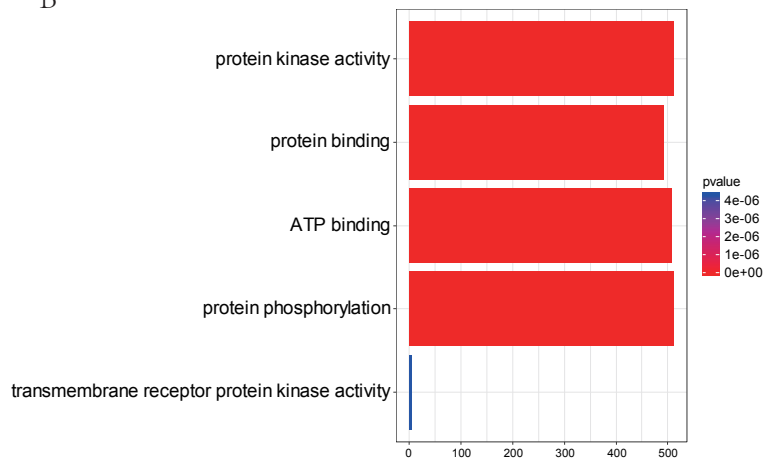

C

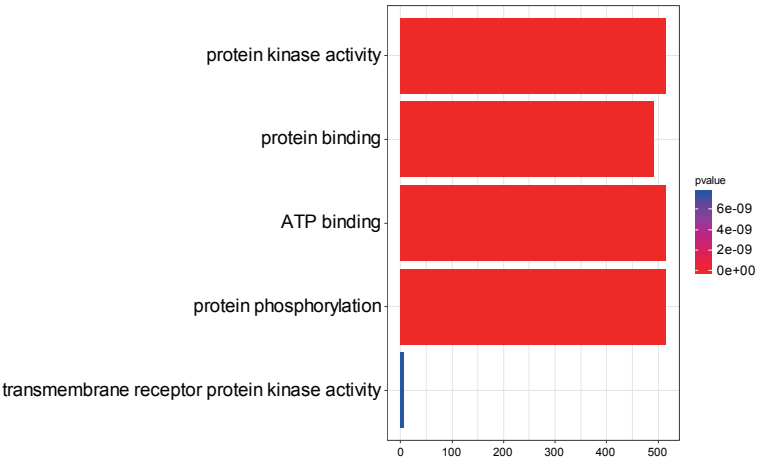

D

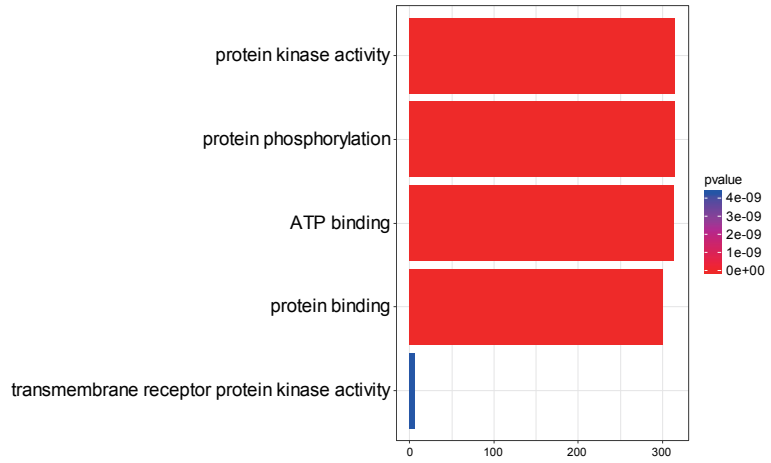

Supplement: Supplementary file 1 [file genes-09-00592-s001.zip › Supplementary Materials (Figure S4).pdf]
