# Supplementary material for: Genome-Wide Analysis of LRR-RLK Gene Family in Four Gossypium Species and Expression Analysis during Cotton Development and Stress Responses
Source: Genes (Basel). 2018 Nov 29;9(12):592. doi: 10.3390/genes9120592 (PMC6316826; doi:10.3390/genes9120592)

Figure S1: Domain and exon-intron organization of identified LRR-RLK family gene members in *A. thaliana* and *Gossypium*.

Exon-intron structures of all identified *LRR-RLK* genes from four *Gossypium* species and *A. thaliana*. LRR, KD and TM domain coding regions were marked on exons by different colored rectangles. 21 subfamilies were distinguished by different color branches.

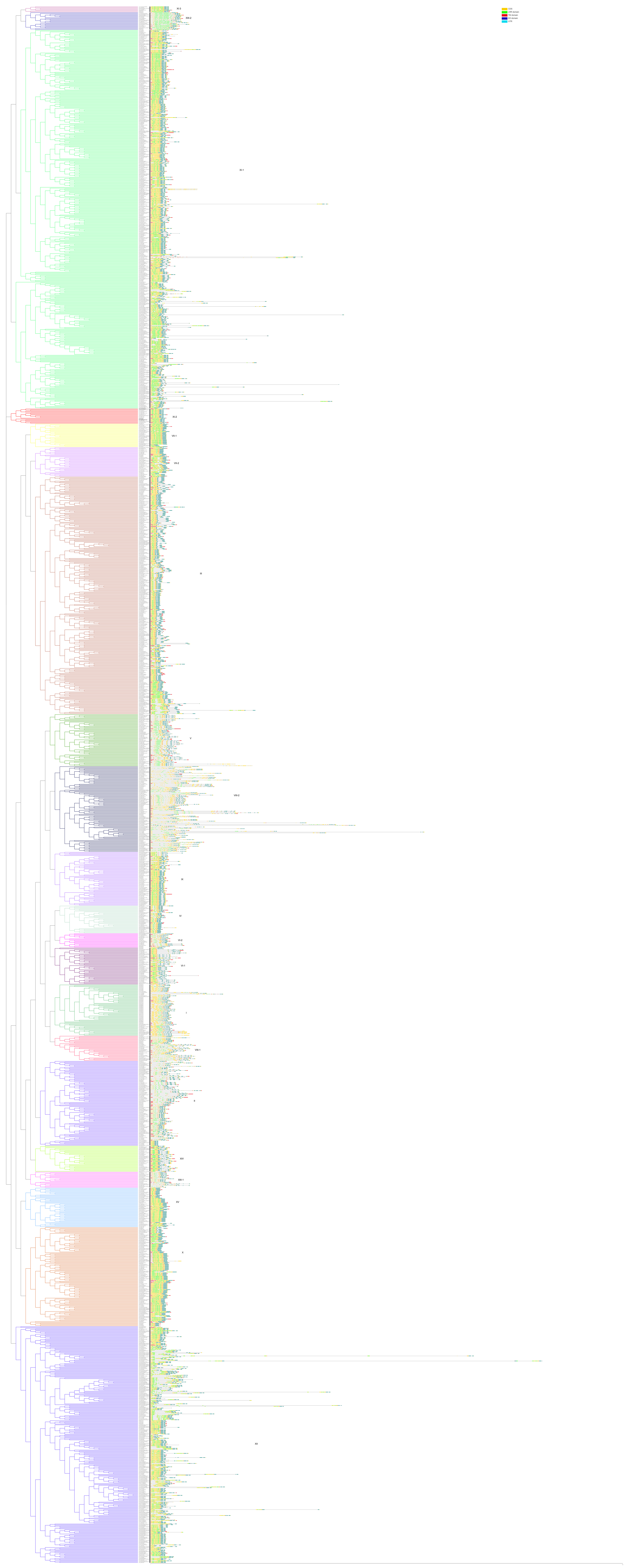

Supplement: Supplementary file 1 [file Supplementary Materials (Figure S1).pdf › Supplementary Materials (Figure S1).pdf]
